# Oxidative Stress in Kidney Injury and Hypertension

**DOI:** 10.3390/antiox13121454

**Published:** 2024-11-27

**Authors:** Willaim J. Arendshorst, Aleksandr E. Vendrov, Nitin Kumar, Santhi K. Ganesh, Nageswara R. Madamanchi

**Affiliations:** 1 Department of Cell Biology and Physiology, University of North Carolina, Chapel Hill, NC 27599, USA; william_arendshorst@med.unc.edu; 2 Department of Internal Medicine, Division of Cardiovascular Medicine, University of Michigan, Ann Arbor, MI 48109, USA; vendrov@med.umich.edu (A.E.V.); kumarni@med.umich.edu (N.K.); sganesh@med.umich.edu (S.K.G.); 3 Department of Human Genetics, University of Michigan, Ann Arbor, MI 48109, USA

**Keywords:** oxidative stress, NADPH oxidases, ROS, mitochondrial dysfunction, redox-sensitive signaling pathways, inflammation, fibrosis, vascular remodeling, hypertension, renal dysfunction, chronic kidney disease, Nrf2/ARE pathway, NF-κB signaling, renin–angiotensin–aldosterone system (RAAS), angiotensin II, TGF-β signaling, antioxidants, SGLT2 inhibitors, MR receptor antagonists

## Abstract

Hypertension (HTN) is a major contributor to kidney damage, leading to conditions such as nephrosclerosis and hypertensive nephropathy, significant causes of chronic kidney disease (CKD) and end-stage renal disease (ESRD). HTN is also a risk factor for stroke and coronary heart disease. Oxidative stress, inflammation, and activation of the renin–angiotensin–aldosterone system (RAAS) play critical roles in causing kidney injury in HTN. Genetic and environmental factors influence the susceptibility to hypertensive renal damage, with African American populations having a higher tendency due to genetic variants. Managing blood pressure (BP) effectively with treatments targeting RAAS activation, oxidative stress, and inflammation is crucial in preventing renal damage and the progression of HTN-related CKD and ESRD. Interactions between genetic and environmental factors impacting kidney function abnormalities are central to HTN development. Animal studies indicate that genetic factors significantly influence BP regulation. Anti-natriuretic mechanisms can reset the pressure–natriuresis relationship, requiring a higher BP to excrete sodium matched to intake. Activation of intrarenal angiotensin II receptors contributes to sodium retention and high BP. In HTN, the gut microbiome can affect BP by influencing energy metabolism and inflammatory pathways. Animal models, such as the spontaneously hypertensive rat and the chronic angiotensin II infusion model, mirror human essential hypertension and highlight the significance of the kidney in HTN pathogenesis. Overproduction of reactive oxygen species (ROS) plays a crucial role in the development and progression of HTN, impacting renal function and BP regulation. Targeting specific NADPH oxidase (NOX) isoforms to inhibit ROS production and enhance antioxidant mechanisms may improve renal structure and function while lowering blood pressure. Therapies like SGLT2 inhibitors and mineralocorticoid receptor antagonists have shown promise in reducing oxidative stress, inflammation, and RAAS activity, offering renal and antihypertensive protection in managing HTN and CKD. This review emphasizes the critical role of NOX in the development and progression of HTN, focusing on its impact on renal function and BP regulation. Effective BP management and targeting oxidative stress, inflammation, and RAAS activation, is crucial in preventing renal damage and the progression of HTN-related CKD and ESRD.

## 1. Introduction

Hypertension (HTN) is a significant contributor to chronic kidney disease (CKD) and end-stage renal disease (ESRD), affecting millions of people around the world. The complex relationship between HTN and renal dysfunction involves a multitude of genetic, environmental, and biochemical factors, with oxidative stress identified as a critical driver of disease progression. Oxidative stress occurs when reactive oxygen species (ROS) production exceeds the body’s antioxidant defenses, resulting in molecular and cellular damage. In the context of HTN-related renal dysfunction, excessive ROS production disrupts critical signaling pathways, alters cellular functions, and contributes to pathological changes in kidney structure.

NADPH oxidase (NOX) enzymes, particularly NOX1, NOX2, and NOX4 isoforms, are significant sources of ROS in the kidney and vascular tissues. Activation of these enzymes under pathophysiological conditions results in the overproduction of ROS, which disrupts redox-sensitive signaling pathways, such as the nuclear factor-kappa B (NF-κB) and transforming growth factor-beta (TGF-β) pathways. These pathways are critical in mediating inflammation, fibrosis, and remodeling of kidney vasculature. ROS damage endothelial cells through these mechanisms, promote inflammatory cytokine production and trigger epithelial-to-mesenchymal transition (EMT), which contributes to renal fibrosis and impaired glomerular filtration.

Furthermore, oxidative stress exacerbates the activity of the renin-angiotensin-aldosterone system (RAAS), a key regulator of blood pressure (BP) and electrolyte balance. Angiotensin II, a central effector of RAAS, stimulates NOX activation and increases ROS production, creating a vicious cycle that heightens oxidative stress, inflammation, and fibrosis. This dysregulated interaction between RAAS-ROS impairs the kidney’s capacity to efficiently excrete sodium, resetting the pressure–natriuresis relationship and perpetuating sustained hypertension. Additionally, antioxidant response pathways such as the Nrf2/ARE are often suppressed in hypertensive states, weakening cellular defenses against oxidative damage and exacerbating renal injury.

Therapeutic strategies that target these molecular pathways—such as RAAS inhibitors, SGLT2 inhibitors, and mineralocorticoid receptor antagonists—offer promise in reducing oxidative stress, enhancing renal function, and slowing the progression of hypertensive kidney damage.

This review examines the molecular pathways through which oxidative stress contributes to renal dysfunction and hypertension. By detailing the interactions between ROS and signaling cascades involved in inflammation, fibrosis, and vascular remodeling, we aim to highlight the potential of targeting oxidative stress pathways for therapeutic interventions. Addressing oxidative stress could not only alleviate hypertensive kidney injury but also provide a broader strategy to combat CKD progression and improve patient outcomes.

## 2. Hypertension and Nephrosclerosis

Hypertension (HTN) affects over 1 billion people, or about 30% of the population worldwide [1]. According to the 2017 American College of Cardiology (ACC)/American Heart Association (AHA) guideline, 46% of American adults have high blood pressure (BP) [2]. Diabetic kidney disease is the leading cause of end-stage renal disease (ESRD) or chronic kidney disease (CKD) worldwide [3]. It accounts for over 50% of individuals entering dialysis or transplant programs in the USA [4]. HTN is a significant risk factor for stroke, coronary heart disease, CKD, and premature death. Approximately 20% of adults with HTN progress to ESRD or CKD. In turn, 80–85% of patients with CKD are hypertensive [5], resulting in impaired renal function, ESRD, severe cardiovascular events, such as coronary heart disease and stroke, and premature death [6]. Approximately one-half of patients with essential HTN have salt-sensitive (SS) HTN [7].

### 2.1. Hypertension Disrupts Renal Structure and Function

A major target organ in HTN is the kidney [8]. Kidney damage occurs as a result of HTN. HTN is the second leading cause of ESRD after diabetes [9]. In patients with HTN and diabetes, circulating oxidative stress and inflammation markers predict renal injury and dysfunction, as reflected by a reduced glomerular filtration rate (GFR) and albuminuria [9]. In ESRD, there are important differences in the incidence of hypertensive fibrosis and renal injury across diverse patient populations, indicating a combination of genetic, environmental, and age-related risk factors contribute to ESRD onset and progression [9]. Activation of the renal angiotensin/aldosterone system (RAAS), Ang II/AT_1_ receptor signaling, the aldosterone/mineralocorticoid receptor (MR) pathway, and the positive relationship between oxidative stress and inflammation are primary interacting pathophysiological mechanisms associated with kidney injury in HTN [10]. NADPH oxidase (NOX)-derived reactive oxygen species (ROS) and oxidative stress contribute to a variety of renal pathologies and fibrosis through the modification of lipids and proteins, the damage of DNA, and the activation of transcriptional factors [8,9,11].

**Figure 1:**
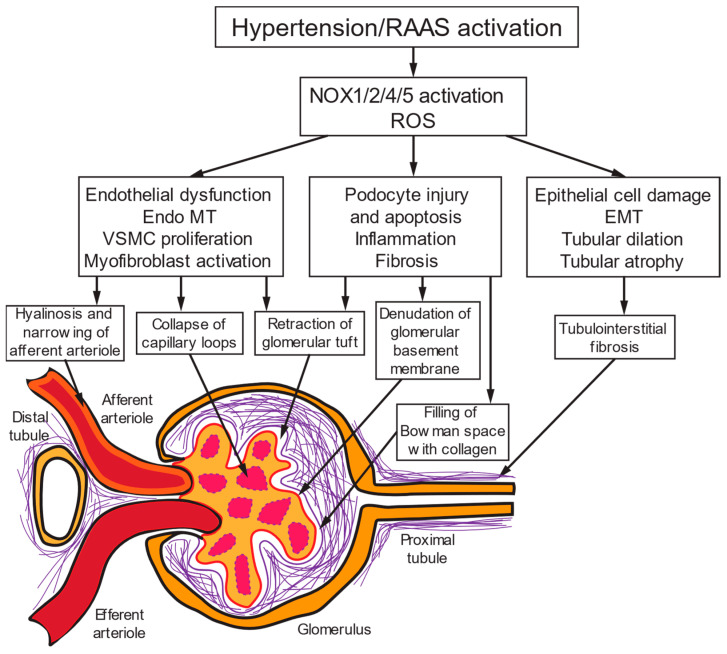
**An intricate network of pathways leads to renal nephrosclerosis in hypertension.** Hypertension and RAAS (renin–angiotensin–aldosterone system) activation initiate a cascade of events, including NOX activation and ROS generation, which induce endothelial dysfunction, inflammation, and epithelial-to-mesenchymal transition (EMT). The resulting podocyte injury and apoptosis contribute to the denudation of the glomerular basement membrane. Concurrently, endothelial-to-mesenchymal transition (EndoMT), vascular smooth muscle cell (VSMC) proliferation, and myofibroblast activation occur, leading to hyalinosis and narrowing of the afferent arteriole, the collapse of capillary loops, and retraction of the glomerular tuft. Epithelial cell damage and EMT lead to tubular dilation, atrophy, inflammation, and fibrosis, ultimately resulting in tubulointerstitial fibrosis and the filling of Bowman’s space with collagen.

Chronic HTN affects renal structure and function, resulting in nephrosclerosis or hypertensive nephropathy, the second leading cause of death in CKD [12]. Chronic HTN results in structural damage of the renal vasculature, glomeruli, and tubular epithelium, with interstitial fibrosis associated with aberrant activation of renal myofibroblasts and excessive generation of extracellular matrix (ECM) proteins such as collagen [13,14]. Myofibroblasts may originate from activated renal fibroblasts, pericytes, epithelial (EMT)-or endothelial to-mesenchymal transition (EndoMT), bone marrow-derived cells, and fibrocytes (Figure 1) [14].

Patients with severe HTN with inadequate blood pressure (BP) control manifest nephrosclerosis with damage to the renal vascular, glomeruli, tubules, and interstitium [15]. Severe systemic HTN and reduced renal mass commonly characterize animal models of progressive renal injury. Weak renal autoregulatory mechanisms and inappropriate responses of preglomerular arteriolar resistance contribute to the complete transmission of elevated BP to intrarenal structures, including baro-sensitive glomeruli [16]. Another cause of glomerular capillary HTN is preferential constriction of efferent arterioles when Ang II levels are high [16]. In part, podocyte dedifferentiation, podocyte-mediated endothelial dysfunction, and podocyte-induced epithelial-mesenchymal transition mediate the complications of barotrauma causing arterial and glomerular hypertrophy, glomerular capillary HTN, and eventual proteinuria [17]. Increased intraglomerular pressure/mechanical strain acts on mesangial cell caveolae and cytoskeleton to activate Rac1/NOX/ROS signaling that activates RhoA [18]. Deranged cellular processes include altered gene expression, tissue damage, sclerosis, fibrosis, apoptosis, and necrosis [19].

Intraglomerular pressure is a primary determinant of the progression of renal failure [20,21]. The CCB benidipine antagonizes L-type Ca^2+^ channels in afferent arterioles and T-type Ca^2+^ channels in efferent arterioles to reduce glomerular capillary pressure and protect from the progression of glomerular barotrauma and glomerulosclerosis in hypertensive patients [22]. The dual L/T-type CCB, manidipine, reduces glomerular capillary pressure more effectively in patients with arterial HTN than the L-type antagonist amlodipine [23].

### 2.2. Oxidative Stress Impairs Renal Function to Promote the Development of Hypertension

Animal models indicate that vascular and renal oxidative stress is an imbalance between oxidants and antioxidants that favors the former [24,25]. Disrupted oxidation-reduction (redox) signaling, combined with renal tubulointerstitial inflammation, is central to abnormal function associated with the pathogenesis of HTN [24,25]. HTN causes oxidative stress and inflammation in renal and cardiovascular tissues [13,24]. Thus, an underlying theme is that oxidative stress, inflammation, and arterial HTN participate in a self-perpetuating vicious cycle that leads to the initiation and progression of cardiovascular and renal disease and most forms of HTN [24]. Additionally, the intrarenal RAAS participates significantly in these pathological processes [26]. For example, local oxidative stress and inflammation impair renovascular function to mediate the initiation and maintenance of Ang II-induced HTN and SS HTN [13]. Functional abnormalities include endothelial dysfunction, exaggerated vasoconstriction, hypoxia, changes in filtration dynamics, a progressive decline in GFR, decreased Na^+^ excretion, and an expansion of extracellular fluid volume, leading to HTN and CKD [12,26]. In summary, exaggerated oxidative stress interacts with inflammation, RAAS, and SNS to cause progressive renal glomerular, tubular, and interstitial injuries in HTN and nephrosclerosis. Arterial, glomerular, and tubulointerstitial injuries occur precipitously during accelerated and malignant HTN [27]. Pharmacological antihypertensives such as RAAS inhibitors slow the progression of renal fibrosis and damage and may allow some recovery during HTN [28].

### 2.3. Reactive Oxygen Species and Cell Signaling

Excessive NOX-derived oxidative stress contributes to multiple renal pathologies and fibrosis through its ability to modify lipids and proteins, damage DNA, and activate transcriptional programs. During chronic renal injury, NOX4 usually protects against interstitial fibrosis and tubular cell apoptosis [29]. NOX1, NOX2, and NOX5 promote renal inflammation and fibrosis by activating ROS-sensitive pathways such as PKC and MAP kinase, proinflammatory cytokines, chemokines, and profibrotic factors [30,31].

By stimulating renal ROS production and suppressing SOD activity, chronic Ang II infusion leads to an increase in myofibroblast infiltration, collagen deposition, and fibrosis, all of which are initiated in the glomeruli and progress to tubulointerstitial structures across the cortex and medulla [32,33]. A chronic NOS/NO inhibition model of HTN in rats shows that activated RAAS (renin–angiotensin–aldosterone system) in the renal cortex is linked to the upregulation of TGF-β1 and fibronectin, which are significant factors producing renal injury (glomerulosclerosis, proteinuria, and interstitial fibrosis) [34,35]. Renal Ang II/AT_1_ receptor stimulation activates TGF-β1, myofibroblast infiltration, collagen accumulation, proximal tubule hypertrophy, and fibrosis [36]. The uremic toxin indoxyl sulfate-induced activation of (pro)renin receptors and prorenin/renin induces expression of TGF-β1 and α-smooth muscle actin via NOX/ROS/Stat3/NF-kB signaling in proximal tubular cells [37].

NOX-derived ROS mediate aldosterone-induced fibrotic signaling. Mineralocorticoid receptor (MR) activation stimulates oxidative stress to cause renal proximal tubular injury during SS HTN [38]. Inhibition of the RAAS by ACE inhibition, AT_1_ receptor blockade (ARB), and MR antagonism is renoprotective in reducing renal injury and suppressing NOX/ROS activity, glomerular capillary pressure, proteinuria, profibrotic TGF-β1 signaling, podocyte abnormalities, glomerular sclerosis inhibit compensatory renal growth [39,40]. Reducing renal monocyte/macrophage-driven inflammation and oxidative stress restores eNOS/NO activity and attenuates HTN and renal injury (glomerulosclerosis, proteinuria, fibrosis) in Ang II-induced HTN [41]. The SOD mimetic tempol reduces ROS-driven MAP kinase/ERK1/2 and JNK activities and ameliorates progressive glomerular sclerosis, proteinuria, and cortical collagen content [42]. Apocynin, an inhibitor of NOX subunit assembly, inhibits NOX activity, expression of NOX2, TGF-β1 expression, ERK activation, macrophage/myofibroblast infiltration, and interstitial fibrosis in kidneys with CKD [43]. Other treatment modes include a diuretic, a low sodium diet (<2 g/day), a low protein diet (~0.7 g/kg/day), α-adrenergic receptor blockers, and Ca^2+^ channel blockers (CCB) [44]. With effective therapy, the kidneys can regenerate and repair tissue injury through autophagy and differentiation of resident embryonic stem cells [45].

Glomerular injury progresses to tubular damage due to tubulointerstitial inflammation and fibrosis [46]. Filtered albumin or circulating glycosylated albumin upregulates NOX4/ROS, TGF-β1, and MCP-1 expression in proximal tubules, resulting in increased urinary H_2_O_2_ levels, interstitial inflammation, and migration of mononuclear cells, fibrosis, and apoptosis [47,48]. A Klotho-derived peptide inhibits TGF-β1/Smad2/3/MAP kinase signaling and associated fibroblast activity to prevent renal fibrosis [49].

In salt-dependent models of HTN (e.g., Dahl SS rats, salt-loaded SHR-SP, DOCA-salt rats, and Ang II-infusion HTN), the production of vasoconstrictors is increased in the endothelium and the kidney [25]. Ang II and ET-1 increase oxidative stress and the inflammatory response in the vascular wall, resulting in endothelial dysfunction and remodeling. AT_1_ and ET-1 receptor antagonism reduces BP in hypertensive patients and vascular hypertrophy in SS HTN patients [50].

Lack of PKC-β limits increased renal cortical expression of NOX2 and NOX4, TGF-β1, VEGF, collagens IV and VI, urinary excretion of 8-isoprostane, and kidney injury in diabetes. Reduced ROS formation is associated with reduced renal hypertrophy, glomerular enlargement, hyperfiltration, and proteinuria [51].

**Figure 2:**
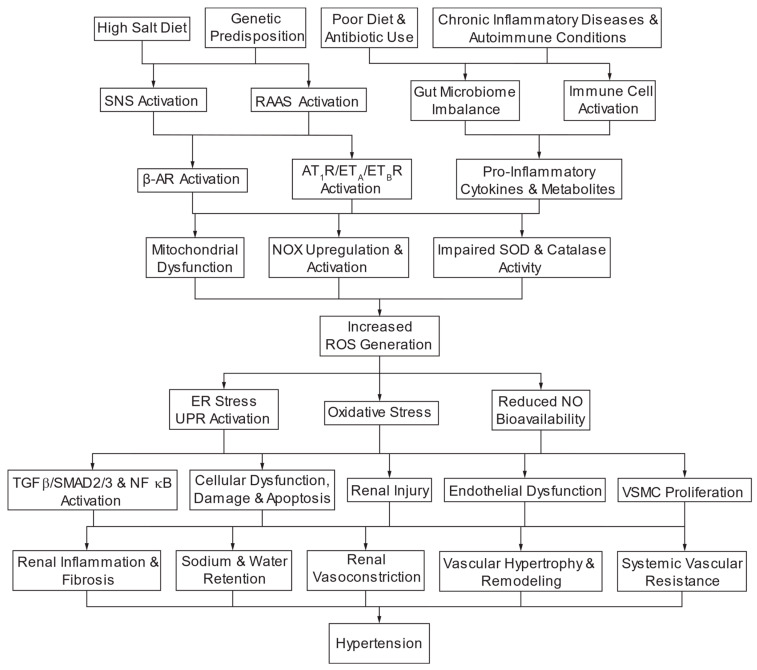
**Oxidative stress is central to the pathophysiological pathways leading to hypertension.** Unhealthy dietary habits and antibiotic use disrupt the gut microbiome, leading to an imbalance that promotes the release of pro-inflammatory cytokines and metabolites and the activation of immune cells. The presence of inflammatory mediators in conjunction with other comorbidities results in reduced nitric oxide (NO) bioavailability and impaired activities of SOD and catalase, inducing mitochondrial dysfunction and increased ROS generation. This, in turn, triggers endoplasmic reticulum (ER) stress and the unfolded protein response (UPR). Critical signaling pathways like TGFβ/SMAD2/3, NFκB, and RAAS) are activated, further worsening oxidative stress by upregulating NOX enzyme levels. Oxidative stress is a pivotal downstream event in the pathophysiological cascade, causing cellular dysfunction, damage, and apoptosis. In the kidney, oxidative stress induces renal inflammation, fibrosis, endothelial dysfunction, and VSMC proliferation. These processes result in renal vasoconstriction, vascular hypertrophy, remodeling, and increased sodium and water retention, leading to elevated systemic vascular resistance and blood pressure, ultimately causing hypertension.

Elevated plasma and urine 8-iso-PGF_2α_ (8-isoprostane) and transforming growth factor β1 (TGF-β1) levels are indicators of impaired renal function in HTN patients with stages 1–5 of CKD [52]. Endothelin (ET-1) and C-reactive protein (CRP) also inversely correlate with GFR [52]. ROS stimulate TGF-β1-induced Smad2/p38 MAP kinase/ERK1/2 activation and fibronectin secretion, EMT, in proximal tubular epithelial cells, culminating in tubulointerstitial fibrosis [19]. In turn, TGF-β1 stimulates NOX-derived ROS with H_2_O_2_ induction of EMT. TGF-β1 stimulates O_2_^•−^ production in the vasculature, decreasing NO-dependent vascular relaxation and remodeling and enhancing contractility [16]. TGF-β1 signaling through Smad2/3 induces mitochondrial NOX4 expression while inhibiting the expression of MnSOD and catalase and stimulating interleukin-6 (IL-6) release from vascular smooth muscle cells (VSMC) (Figure 2) [53]. NOX4 overexpression and O_2_^•−^ generation activates p38 MAP kinase in endothelial cells [18]. In podocytes, TGF-β1 induces Smad2/3 activation of NOX4/ROS, fibrosis, and caspase-3-mediated apoptosis and detachment associated with increased glomerular permeability to albumin [54]. However, another study reports that overexpression of NOX4 in proximal tubular cells does not modify the course of fibrosis in CKD [55]. Hyperglycemia activates NOX1 and ROS production, which participates in mesangial fibrogenesis via iNOS induction.

### 2.4. Summary

HTN contributes to kidney damage, leading to conditions such as nephrosclerosis and hypertensive nephropathy, which are significant causes of CKD and ESRD. HTN also is a risk factor for stroke and coronary heart disease. Oxidative stress, inflammation, and RAAS activation are fundamental pathophysiological mechanisms causing kidney injury in HTN. These factors lead to a vicious cycle, exacerbating renal and cardiovascular disease progression. Genetic and environmental factors influence the susceptibility to hypertensive renal damage. African American populations, for example, have a higher tendency to develop severe nephrosclerosis and SS HTN compared to Caucasian patients, potentially due to genetic variants. Adequate BP management with treatments targeting RAAS activation, oxidative stress, and inflammation is critical to preventing renal damage and the progression of HTN-related CKD and ESRD. Pharmacological interventions, such as antioxidants, RAAS inhibitors, dietary changes, and novel drugs aimed at specific genetic pathways for individualized treatment, may be beneficial in reducing and possibly reversing kidney damage in hypertensive patients.

## 3. Renal Mechanisms Contributing to the Development of Hypertension

The etiology of HTN involves multiple interactions among genetic and environmental factors impacting pathological mechanisms and physiological homeostatic regulatory systems. Oxidative stress and elevated ROS, upregulation of the RAAS, activation of the SNS, and inflammation, often combined with reduced NO, play significant roles in developing and maintaining HTN [56,57].

Renal cross-transplantation studies convincingly establish that a primary abnormality in kidney function is critical to the development of HTN in different animal models, including spontaneously hypertensive rats (SHR) and stroke-prone SHRs (SHR-SP), Milan hypertensive rats, Dahl SS rats, and Ang II-induced HTN [58,59]. Post-transplantation HTN in a kidney recipient develops when the host is hypertensive-prone but not when the host is normotensive [59]. Supportive evidence comes from metabolic balance and renal function studies showing that Na^+^ retention is exaggerated during the developmental phase of HTN. In contrast, salt balance is maintained after HTN has been established [60,61]. A transient phase of reduced Na^+^ excretion resulted from reduced GFR, filtered Na^+^ load, and enhanced tubular reabsorption [62]. Human renal transplant studies also show that the genetic background of the kidney donor with a predisposition for HTN significantly influences the recipient’s BP and the need for antihypertensive therapy [63].

### 3.1. Resetting of the Pressure–Natriuresis Relation

**Figure 3:**
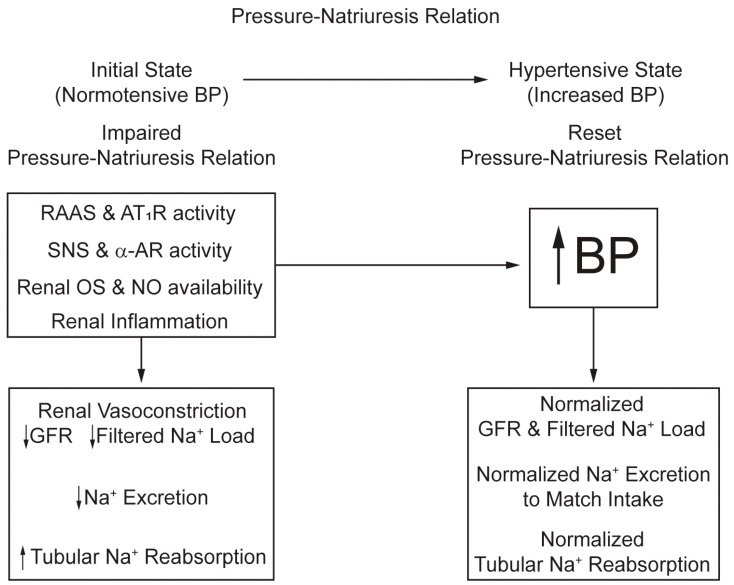
**Mechanisms affecting renal function and pressure–natriuresis relation in the development of hypertension.** The pressure–natriuresis relationship describes the kidney’s ability to excrete Na^+^ in response to changes in BP. Increased SNS and RAAS activity, renal oxidative stress, and inflammation at normotensive BP result in impaired pressure–natriuresis, characterized by reduced Na^+^ excretion. That leads to elevated BP and a “reset” of the pressure–natriuresis curve, normalizing Na^+^ excretion to match sodium intake levels. This normalization is characterized by improved Na^+^ filtration capacity and reduced Na^+^ retention. Up arrows indicate an increased effect, while down arrows signify a decreased effect.

Mechanisms that reset the slope and intercept of the pressure–natriuresis relation with anti-natriuretic actions include increased Ang II/AT_1_ receptor and sympathetic nerve activity, ROS levels, and inflammation, and reduced NO exerts such anti-natriuretic actions (Figure 3) [64]. Evidence supports several non-exclusive views regarding the anti-natriuretic effects of increased ROS and inflammation [65]. Key factors include decreased RBF and GFR, intrarenal NO, intrarenal hydrostatic pressure or medullary blood flow, renal AT_2_ receptor density combined with increased Ang II/AT_1_ receptor stimulation, and renal sympathetic nerve activity [66,67,68]. Renal endothelial dysfunction is manifested as a decrease in the release of vasodilatory mediators such as NO, prostacyclin, and hyperpolarizing factors, and an increase in vasoconstrictor mediators such as Ang II, endothelin (ET-1), and thromboxane (TxA_2_) [60]. In VSMC, increased intracellular Ca^2+^ concentration, PKC, Rho kinase, and MAPK promote vasoconstriction [69]. Matrix metalloproteinases and their inhibitors modify the ECM composition and lead to renal vasculature remodeling [70].

Various studies in humans and experimental animals show that the overproduction of ROS and impaired antioxidant mechanisms play a central role in the development of HTN. Genetic animal studies demonstrate that deleting specific ROS-producing NOX isoforms reduces vascular and renal ROS production and prevents or alleviates HTN [25]. Similarly, the genetic deletion of superoxide dismutase (SOD) isoforms resulted in increased ROS levels and increased BP [71].

### 3.2. Importance of the Intrarenal Renin-Angiotensin System and Endothelin

The RAAS, especially the intrarenal RAS, plays a significant role in developing and maintaining HTN in experimental models, including SHR, Ang II-induced HTN, and Dahl SS HTN [72]. AT_1_ receptors are upregulated in the proximal tubule. Both circulating and intrarenal Ang II stimulates intrarenal renin to magnify the amount of intrarenal Ang II, a vasoconstrictor and activator of Na^+^ transporters along the nephron, thus promoting Na^+^ retention and expansion of extracellular fluid volume, and raising BP [73,74]. Intrarenal Ang II/AT1 receptor activation and reduced NO bioavailability shift the pressure–natriuresis relation to the right, exerting an anti-natriuretic action requiring a higher perfusion pressure to normalize Na^+^ excretion to match intake [75]. Although Ang II downregulates AT_1_ receptor density in VSMC, Ang (1–7) increases AT_1_ receptor mRNA expression [73,76]. Ang II acts as a growth factor leading to vascular hypertrophy independent of changes in BP, leading to elevated vascular resistance [77]. The potentiating effects of Ang II are achieved by the AT1 receptor upregulation of NOX expression and activity, ROS generation, and inflammation, which elicit vasoconstrictor and anti-natriuretic effects [78]. Ang II actions are also potentiated by AT_1_ receptor-mediated suppression of NO and antioxidant systems [79]. ACE inhibitors and ARBs reduce BP in previously hypertensive animals and prevent the development of HTN [80,81]. Furthermore, genetic deletion of renal ACE protects against the development of Ang II-induced HTN [74].

ET-1 production by the kidney and vasculature also contributes to developing and maintaining experimental HTN. The renal medulla is rich in ET-1, regulates BP and Na^+^ excretion by reducing local blood flow primarily via ET_A_ receptors, and mediates salt and water reabsorption by the TAL and collecting duct through ET_B_ receptors [82,83]. There is increased prepro-ET-1 mRNA or peptide expression in the vasculature and kidneys of hypertensive patients, SHR, Ang II-induced HTN, and Dahl SS HTN [50]. Major stimuli of ET-1 formation include ROS and Ang II. Ang II-induced renal vasoconstriction involves ET-1-mediated stimulation of TxA_2_ formation in SHR [84]. ET-1 is also essential in oxidative stress, inflammation, and resetting the pressure–natriuresis relation, increasing Na^+^ retention at perfusion pressure, glomerulosclerosis, and renal damage [85]. ET_A_ receptors cause vasoconstriction, endothelial dysfunction, vascular growth and hypertrophy, glomerulosclerosis, and salt sensitivity with pro-oxidative and pro-inflammatory actions [86]. ET-1/ET_A_/ET_B_ receptor-induced O_2_^•−^ production stimulates overexpression of G_i_α proteins in SHR VSMC by activating Src/PDGF/MAPK signaling [87]. Transgenic mice overexpressing ET-1 develop glomerulosclerosis, interstitial fibrosis, and renal cysts but not HTN [88]. Dual ET_A_/ET_B_ and ET_A_ receptor antagonists reduce BP, vascular reactivity, and renal injury in hypertensive SHR, the Ang II infusion model, and Dahl SS rats [89]. ET_B_ receptors stimulate NO production to cause renal vasodilation [90]. Selective ET_B_ receptor blockade causes endothelial dysfunction and reduces RBF and GFR in adult SHRs [91].

### 3.3. Gut Microbiota

The abundance of the gut microbes, firmicutes, and Bacteroidetes is associated with increased BP in models of genetic HTN, such as SHR and Dahl SS rats [92]. A reduction in gut microbiota caused by antibiotics can increase or decrease BP depending on the genotype affected [93]. Fermentation of nutrients by gut microbiota produces metabolites that can affect BP by regulating energy expenditure, intestinal catecholamine metabolism, epithelial ion transport, and, thus, salt sensitivity [94]. The colonic epithelium produces and transports many microbial vasoactive metabolites and neurotransmitters that regulate the immune system, oxidative stress, and BP [95]. Key metabolites are butyrate, indole, tryptophan, and short-chain fatty acids (SCFAs) that target NOX4/ROS and pro-inflammatory TNF-α/NF-kB [96]. T-cell activation in the gut immune system and T-cell accumulation in the vasculature contribute to gut microbiota modulation of BP [97]. Gut microbiota modulation leading to changes in butyrate absorption may impact the effectiveness of antihypertensive treatments, as seen in studies with ester ACE inhibitors [98]. Additionally, manipulating gut microbiota by MR blockade may improve gut dysbiosis and reduce gut sympathetic tone, suggesting potential therapeutic avenues for managing HTN [99].

### 3.4. Summary

Interactions between genetic and environmental factors leading to kidney function abnormalities are central to the development of HTN. Animal renal cross-transplantation studies suggest that the genetic background of the kidney donor can significantly influence the recipient’s BP. Anti-natriuretic mechanisms—such as SNS activation, increased Ang II, ROS, inflammation, or reduced NO—reset the pressure–natriuresis relationship, necessitating higher BP to excrete Na^+^ properly (Figure 3). Intrarenal Ang II/AT1 receptor activation increases Na^+^ retention and BP by upregulating ROS, while ET-1 contributes to HTN by causing oxidative stress, inflammation, vasoconstriction, endothelial dysfunction, and Na^+^ excretion. The gut microbiome, mainly firmicutes and Bacteroidetes, is associated with HTN and can affect BP by influencing energy expenditure, salt sensitivity, and inflammatory pathways by producing vasoactive metabolites. Drugs that target Ang II, AT1, or ET receptors can alleviate oxidative stress and renal injury and prevent or reduce HTN.

## 4. NADPH Oxidases and Reactive Oxygen Species in the Kidney

### 4.1. Normotensive Animals

**Figure 4:**
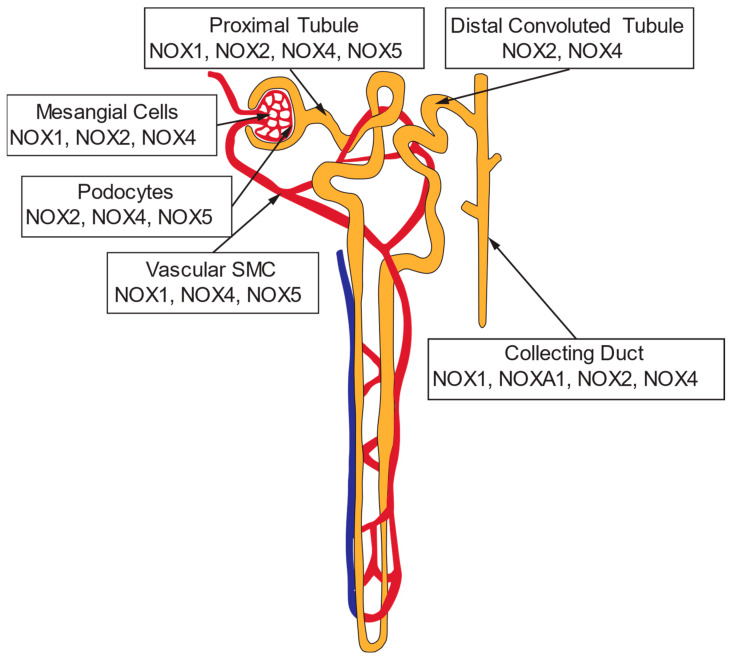
**NADPH oxidases expression in the renal cells.** NADPH oxidases (NOX) and mitochondria are the primary sources of ROS in renal and vascular cells, including VSMC, podocytes, mesangial cells, and tubular epithelial cells.

A controlled balance of ROS and antioxidant systems in health primarily involves O_2_^•−^, H_2_O_2_, and NO [100]. Such balance is essential in regulating signaling pathways and maintaining renal structure and function [101]. ROS are mainly produced by cell membrane nicotinamide adenine dinucleotide phosphate (NADPH) oxidases (NOXs) in response to hormones and physical factors [100]. Other sources of ROS include redox-sensitive mitochondria, endoplasmic reticulum, and uncoupled NOS (Figure 2) [102]. In the vasculature, NOX2 and NOX4 are expressed in endothelial cells, while NOX1, NOX2, and NOX4 are present in rodents’ vascular smooth muscle cells (VSMC) [103]. NOX5 is expressed in humans but not rodents [104]. In the kidney, NOXs produce ROS in endothelial, VSMC, mesangial, podocyte, tubular cells, and fibroblasts [101]. Mitochondria and iNOS also generate ROS [105]. NOX1, NOX2, and NOX4 (and human NOX5) are expressed in the kidney, with a prominent expression in renal vessels, glomeruli, podocytes, and cells of the thick ascending limb of the loop of Henle (TAL), macula densa, distal tubules, collecting ducts, and cortical interstitial fibroblasts (Figure 4) [100,101]. Antioxidant systems protecting against excess ROS and oxidative stress include SODs and catalase, the glutathione and thioredoxin systems [102]. Oxidative stress, excess ROS (O_2_^•−^ and H_2_O_2_)^,^ dominates over NO to affect cell metabolism and cardiorenal function, ultimately causing cell injury and death [102].

NOX1, NOX2, and NOX5 primarily catalyze the reduction of O_2_ to produce O_2_^•−^ [106]. NOX4 produces large amount of H_2_O_2_ constitutively, and its activation does not require other cytosolic oxidase components [107]. All NOXs transfer electrons from NADPH to molecular oxygen, reducing O_2_ to O_2_^•−^ and attendant other downstream ROS, with NADP^+^ being a byproduct [8]. H_2_O_2_ is a principal ROS second messenger signaling molecule acting on downstream pathways [32,108]. Low physiological levels of ROS participate in pro-survival signaling, cell proliferation, and growth [109]. The balance between ROS and NO is crucial in regulating vasomotor tone and salt and water epithelial transport [110]. Downstream pathways include transcription factors, tyrosine kinases/phosphatases, mitogenic factors, and cytokines mediating hormonal effects, regulation of ion channel activity, oxygen sensing, gene expression, cell differentiation, senescence, and apoptosis [8,111]. NOS isoforms generate reactive nitrogen species (RNS) [32]. A imbalance in ROS production and depletion of NO determines the magnitude of oxidative stress. This imbalance promotes inflammation and contributes to the progression of prohypertensive renal disease, and causes tissue damage and fibrosis. These effects occur because ROS and RNS can modify lipids and proteins, damage DNA, and activate transcriptional programs, ultimately leading to cell apoptosis and necrosis [71,110]. The deleterious actions of ROS and RNS are countered by cellular antioxidant defense systems (SODs, catalase, peroxidases, glutathione and thioredoxin systems, and antioxidant vitamins) [112].

Studies in humans and experimental animals show that unrestricted ROS and/or RNS production and impaired antioxidant systems play a central role in HTN during its developmental and established phases [113]. Oxidative stress, elevated ROS, and reduced NO are critical to developing and maintaining HTN in experimental models such as SHR, Ang II-induced, and Dahl SS HTN [110,113]. A positive interaction between oxidative stress and the immune system participates in both phases of HTN [114]. The combination of HTN and diabetes exacerbates oxidative stress in the kidney [115]. Genetic deletion of specific ROS-generating NOX isoforms reduces vascular and/or renal ROS production and prevents or mitigates the development of HTN [113]. Inhibition of ROS with NOX assembly inhibitors and antioxidants, SOD mimetics, blockers of the RAAS, or genetic deletion of one of the components of the signaling cascade usually preserves renal structure and function. It attenuates or delays the onset of HTN. Transgenic rodent studies indicate that global NOX1, NOX2, or NOX4 overexpression promotes HTN. Moreover, genetic deletion of SOD isoforms and other antioxidants increases ROS bioavailability, elevating BP [110]. Pharmacological inhibition of SOD increases renal levels of O_2_^•−^, causing vasoconstrictor and anti-natriuretic responses. These effects are amplified during NOS inhibition, indicating offsetting actions of O_2_^•−^ and NO in regulating renal vascular tone and Na^+^ excretion [110].

Oxidative stress in normotensive animals prone to high BP usually precedes the development of HTN, while reducing excess ROS lowers BP in nearly all HTN models [8,71]. Excess ROS, directly and indirectly, affect various aspects of renal function, including renal hemodynamics and vascular reactivity and remodeling, endothelial dysfunction, glomerular filtration, and filtered Na^+^ load and tubular transport [71]. Such actions interact to regulate the urinary excretion of salt and water and, ultimately, BP. Also activated are regulatory factors such as inflammation, the RAAS, particularly the intrarenal RAS, and sympathetic nerve activity [116]. Overall, renal oxidative stress causes HTN by promoting Na^+^ retention due to enhanced renal vascular resistance, increased tubular Na^+^ transporter activity, and Na^+^ reabsorption primarily localized to the TAL and/or the distal nephron [71]. Excess ROS resets the pressure–natriuresis relation such that HTN-prone animals transiently excrete less Na^+^, leading to Na^+^ retention and increasing BP. Subsequently, elevated renal perfusion pressure normalizes excretion to match the dietary Na^+^ intake [117]. Ang II and a high-salt diet are major stimuli of NOX-derived O_2_^•−^ production and oxidative stress [8]. Resetting the pressure–natriuresis relation leads to the development of HTN, whether induced by Ang II or in genetic models such as SHR and Dahl SS HTN. Inhibition of ROS production and treatment with the O_2_^•−^ scavenger tempol reduce BP and improve renal hemodynamics and excretory function in experimental models of HTN under consideration in this review [13].

Rac1 is a small GTPase essential for NOX enzyme assembly and agonist activation. Agonist stimulation of NOX1, NOX2, and NOX4 involves direct interaction with p22phox [118]. NOX1 and NOX2 require the association with cytosolic regulatory subunits for activity, whereas NOX4 is constitutively active [119]. Catalytic regulatory subunits for NOX2 are p22phox, p47phox, and p67phox, and Rac-1 is activated by WNT signaling. The subunits for NOX1 are NOXO1 organizer protein and NOXA1 activator protein, homologs of p47phox and p67phox, respectively [120]. The constitutive activity of NOX4 is primarily regulated by expression, with activity enhanced by the polymerase (DNA-directed) interacting protein 2 (Poldip2), a p22phox binding partner [121]. NOX5 differs from other NOXs in that it is activated directly by cytosolic Ca^2+^ to produce O_2_^•−^ and does not require cytosolic subunits, including p22phox, for activation. Knockout of p22phox leads to loss of NOX1 and NOX4 activity, but not that of NOX5 [122].

Transgenic mice overexpressing NOXs in the vascular wall have exaggerated vascular and BP responses to Ang II administration [111]. Vascular NOX enzymes and ROS products are linked to cardiovascular pathologies, including cell proliferation and differentiation, angiogenesis, inflammation, hypertrophy and fibrosis, atherosclerosis, and HTN. NOX1, NOX2, and NOX4 (NOX5 in humans) are expressed in vascular endothelial and smooth muscle cells of rodents [123]. In general, O_2_^•−^ production by plasma membrane NOX1, NOX2, and NOX5 is pro-hypertensive by reducing NO bioavailability and promoting eNOS uncoupling, endothelial dysfunction, inflammation, transactivating epidermal growth factor receptor, cell proliferation/migration and apoptosis. In contrast, anti-hypertensive, protective endoplasmic reticulum NOX4 production of H_2_O_2_ increases NO bioavailability and suppresses cell death pathways [8,124].

NOX1/O_2_^•−^, but not NOX2 or NOX4, upregulate arterial T-type Ca^2+^ channel function following chronic NO deficit [125]. Vascular endothelial growth factor (VEGF) protects against vascular injury by increasing eNOS activity and inhibiting NOX1/O_2_^•−^. This is combined with increased nuclear factor erythroid 2-related factor 2 (Nrf2) expression and enhanced antioxidant activity, improved vasodilation and vascular structural remodeling [126]. EGF activates the Rac-1/NOXO1/NOX1 complex to produce O_2_^•−^. Thrombin-induced upregulation of NOX1/O_2_^•−^ is responsible for increased cytosolic Ca^2+^ and proliferation/migration of VSMC [127]. NOX1/ROS promote vascular Kv1.5 protein expression and cytosolic K^+^ levels, remodeling, and apoptosis [123]. Animal studies have shown that oral G-protein coupled estrogen receptor inhibitors act as selective NOX1 protein downregulators, preventing vascular disease, HTN, and glomerular injury [128].

Nrf2 provides renoprotection against oxidative stress- and inflammation-related changes in renal function and injury [129]. Excess ROS activates the Kelch-like ECH-associated protein (Keap1)/Nrf2 pathway, which transcribes antioxidant and cytoprotective genes such as SOD, catalase, and glutathione peroxidase. This activity helps reduce oxidative stress, generate NO, and inhibit ADMA, which inhibits NOS [130]. Patients with CKD exhibit oxidative stress, inflammation, macroalbuminuria, and plasma lipids associated with PPARγ activation. This leads to NF-kB and Wnt/β-catenin signaling activation, while the Nrf2 pathway is downregulated [131]. Redox-sensitive Nrf2 prevents HTN, endothelial dysfunction, increased ADMA, microvascular oxidative stress, and exaggerated reactivity during chronic Ang II infusion [132]. Nrf2 signaling attenuates EMT and renal interstitial fibrosis by inactivating PI3K/Akt signaling [133]. HIF-1 activation in response to hypoxia in renal tubular cells is mediated by downregulation of Nrf2 in mitochondria, with reoxygenation restoring Nrf2 [134]. Nrf2 protects against oxidative stress by reducing proinflammatory cytokine and chemokine expression during ischemic and nephrotoxic kidney injury [129]. Hydrogen sulfide (H_2_S) is renal protective, stimulating the Keap1/Nrf2 pathway to inhibit oxidative stress and associated renal injury (reduced GFR, proteinuria, collagen deposition) induced by high salt in Dahl SS rats [135]. Nrf2 inhibition of Node-like receptor containing pyrin domain-3 (NLRP3) signaling also attenuates the inflammatory response and cell apoptosis [136].

NOX4 is cytoprotective in renal tubular cells, endothelial cells, and VSMC and regulates metabolism when such cells are injured [137]. Mice with NOX4 deletion are more susceptible to acute and chronic tubular injury. NOX4/ROS can downregulate the antioxidant NRF2/HO1 pathway and increase TGF-β1, differentiation of fibroblasts into myofibroblasts, fibronectin and collagen IV expression, tubular cell apoptosis, and renal interstitial fibrosis [138]. Hyperglycemia induces NOX4, p22phox, p47phox, but not NOX1 or NOX2, selectively in the renal cortex. This leads to the activation of p38 MAP kinase and expression of TGF-β1 and fibronectin [139].

NOX5 generation of O_2_^•−^ is upregulated by Ang II, ET-1, and TNF-α in the vasculature and kidneys, particularly in glomerular and proximal tubular cells of patients with HTN and diabetic nephropathy [140,141]. Increased NOX5 expression contributes to proteinuria, renal inflammation, and fibrosis in patients with diabetes [30]. In hypertensive patients, endothelial cells have elevated NOX5/ROS levels, which lead to eNOS uncoupling, promoting additional O_2_^•−^ production and causing endothelial dysfunction [142]. A genome-wide association study identified NOX5 as a BP-associated gene [143]. Transgenic mice that express human NOX5 suggest a pathogenic role for NOX5 in the kidneys, showing glomerular and tubular abnormalities that resemble CKD [144]. Cell-specific NOX5 overexpression in the endothelium, glomerular podocytes, or renal proximal tubular cells elevates resting BP and renal inflammation [144]. On the other hand, the expression of NOX5/ROS in VSMC does not impact BP or vascular structure. However, PDGF stimulation of NOX5 promotes VSMC proliferation mediated by JAK/STAT signaling [145]. VSMC Ca^2+^ levels increase, leading to increased vasoconstriction in response to certain stimuli. Simultaneously, the ability of the blood vessel to dilate in response to the endothelium is reduced [146]. NOX5/O_2_^•−^ mediates basic fibroblast growth factor-induced upregulation of intermediate conductance Ca^2+^-activated K^+^ channels (KCNN4) in VSMC [147].

Elevated plasma ET-1, 8-iso-PGF_2α_ (F2-isoprostane), and TGF-β are predictors of renal dysfunction and reduced GFR in hypertensive patients [52]. ET-1 is a pro-hypertensive vasoconstrictor agent that elicits oxidative stress and inflammation in the vasculature and the kidney [50]. ET-1 stimulates NOX1 and NOX2-derived O_2_^•−^ via ET_A_ receptors, whereas protective ET_B_ receptors are linked to NO and inhibition of NOX1 to reduce O_2_^•−^ production [148]. Oxidative stress and F2-isoprostane/TP receptor stimulate endothelial cell production of ET-1 [149]. In VSMC ET-1/ET_A_ receptor activates NOX-derived O_2_^•−^ to stimulate ERK1/2/PKB/Pyk2 and JAK2 signaling [150,151]. Blocking ET_A_ receptors is reno-protective.

Hyperglycemia produces oxidative stress, upregulating endothelial NOX1/O_2_^•−^, but not NOX2, mediated reduction in NO bioavailability, eNOS uncoupling and endothelial dysfunction, and generation of pro-inflammatory/pro-fibrotic factors [152]. Another report shows hyperglycemia upregulates NOX1, NOX2, NOX4, and iNOS expression in VSMC and cell migration [153]. iNOS is a source of ROS in the kidney. Blockade of Rac-1 activation and siRNA knockdown of NOX1 or NOX4 in VSMC reduce ROS production, improving NO’s ability to inhibit VSMC migration [153]. On the other hand, NO suppresses O_2_^•−^ production by NOX2 and NOX4 in endothelial cells by causing S-nitrosylation of p47phox [154]. Elevated vascular NOX1 O_2_^•−^ production, pro-inflammatory and pro-fibrotic markers, and macrophage infiltration lead to atherosclerosis [155].

Resistant hypertension (RH) is when a patient has elevated BP that remains above the target levels despite using three different types of antihypertensive medications, a long-acting Ca^2+^ channel blocker (CCB), AT_1_ receptor blockade (ARB), and a diuretic, administered at their maximum or highest tolerated doses [156]. Sometimes, patients need four or more medications to reach their target BP. To diagnose RH, it is essential to ensure that the patient is taking their medications as prescribed and to rule out the possibility of a “white-coat effect”, where a patient’s BP is higher in a medical setting than outside the doctor’s office. The management of resistant HTN involves maximizing lifestyle changes, using long-acting thiazide diuretics (chlorthalidone or indapamide), adding MR antagonist (spironolactone or eplerenone), and gradually introducing additional antihypertensive medications with different mechanisms of action if the BP remains high [156,157].

### 4.2. Spontaneously Hypertensive Rats

#### 4.2.1. The Model

The SHR model of genetic HTN replicates many characteristics of essential HTN in humans [158]. Inbred SHR of the Okamoto-Aoki strain are born normotensive and develop HTN after 4–6 weeks of age while consuming a regular salt diet [159]. Established HTN tends to plateau following a progressive rise in BP between 8–14 weeks of age [160]. Renal cross-transplantation studies demonstrate that abnormal kidney function is central to developing genetic HTN in SHRs and stroke-prone SHRs (SHR-SP) [161]. When the donated kidney originates from SHR, post-transplantation HTN develops in age-matched bilaterally nephrectomized F1 hybrids bred from SHR and WKY parents [162]. In contrast, BP does not change in the recipients of donated kidneys from WKY control rats [163]. After post-surgical stabilization, renal function (RBF, GFR) and kidney weight are similar in the two groups. No histological differences are noted except structural vascular hypertrophy in SHR donor kidneys [160,161].

Abnormal renal function in young SHR is primarily due to increased RAAS activity, renal oxidative stress, and interstitial inflammation, all of which induce exaggerated salt retention relative to salt intake at a normotensive BP [164,165]. The pressure–natriuresis relation is shifted to the right as BP becomes elevated to normalize Na^+^ excretion [166,167]. Thus, young SHR excrete less Na^+^ and water during prehypertensive than their WKY normotensive controls fed a regular diet [168,169]. Therefore, an increase in BP is required to normalize Na^+^ retention to match intake. Specific activation of the intrarenal RAS is central to the development and maintenance of genetic HTN in SHR [72,170]. The development of HTN can be prevented by reducing renal RAS activity through ACE inhibition of AT_1_ receptor blockade using ARBs [171,172].

Crossbreeding of young prehypertensive SHR and normotensive WKY reveals that renal vasoconstriction co-segregates with the development of elevated BP in the offspring [173]. Decreased papillary vasa recta blood flow in young prehypertensive SHR may contribute to resetting the pressure–natriuresis relation with enhanced NaCl reabsorption in medullary nephron segments [174]. Adult SHR with established HTN attain normotensive WKY levels of RBF, GFR, and Na^+^ excretion [175]. Oxidative stress, impaired NO activity, and inflammation contribute to abnormal pressure–natriuresis [176]. Longitudinal antioxidant treatment initiated in prehypertensive SHR alleviates vascular oxidant stress and prevents BP elevation [174].

#### 4.2.2. Oxidative Stress in the Kidney

Vascular oxidative stress precedes the development of HTN in young SHR [177]. VSMC NOXs are regulated differently in hypertensive SHR and normotensive WKY rats [178]. In VSMC of SHR, plasma membrane NOX1 and endoplasmic reticulum NOX4 produce O_2_^•−^ and H_2_O_2_ that promote exaggerated contractility and proliferation [179]. Basal and Ang II/AT_1_ receptor-induced NOX1-driven ROS generation are enhanced in VSMC of resistance arteries of 9-week-old SHR developing HTN but not in VSMC of younger prehypertensive rats [180,181]. Mesenteric resistance arteries show a progressive increase in NOX1 and NOX4 mRNA and ROS production in SHR between 8 and 12 weeks of age [181]. During this period, ARB reduced ROS production and BP. Cultured immortalized proximal tubular cells of SHR have elevated basal levels of NOX2, NOX4, and ROS production with reduced protein levels of SOD2, SOD3, and catalase [182].

**Figure 5:**
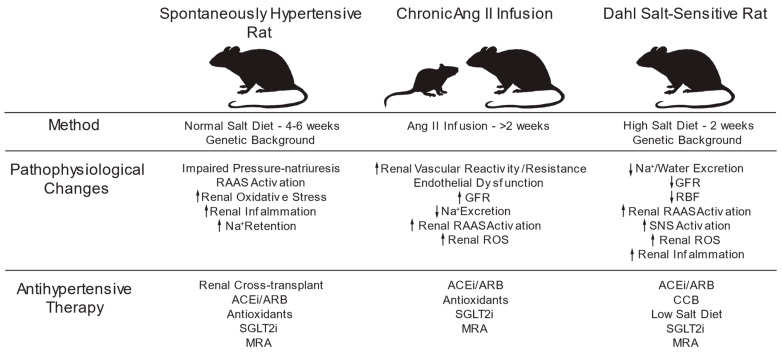
**Animal models of hypertension.** In the Dahl salt-sensitive rat model, a high salt diet leads to renal inflammation, increased ROS levels, activation of the SNS and RAAS, decreased GFR, and changes in Na^+^ and water excretion. The chronic infusion of Ang II leads to similar pathophysiological changes, including increased ROS levels, RAAS activation, impaired Na^+^ excretion, reduced GFR, endothelial dysfunction, and heightened renal vascular reactivity and resistance. In the spontaneously hypertensive rat model, HTN develops through genetic predisposition when the animals are fed a normal salt diet. Pathophysiological changes involve renal inflammation, oxidative stress, RAAS activation, Na^+^ retention, and impaired pressure–natriuresis. Preventive measures across all models include RAAS inhibition (ACEi/ARB), antioxidants, and dietary modifications such as a low or normal salt diet.

The subcellular localization of NOX4 in the endoplasmic reticulum was increased in VSMC from SHR, which was linked to endoplasmic reticulum (ER) stress [179]. Furthermore, VSMC protein hyperoxidation is correlated with oxidative and ER stress, driven by elevated levels of NOX1 in the cell membrane and NOX4 in the endoplasmic reticulum. The intricate interaction between oxidative stress and endoplasmic reticulum stress triggers the unfolded protein response (UPR), leading to increased VSMC proliferation and vascular dysfunction in HTN (Figure 5). Supporting the role of ER stress in regulating hypertension, inhibition of ER stress in Ang II-induced hypertension led to a 20 mmHg decrease in BP [183].

Chronic tempol treatment prevents the BP increase in SHR between 6 and 11 weeks of age in association with reduced vascular production of O_2_^•−^ [177]. Aortic VSMC from 12-week-old SHR exhibit enhanced NOX4 protein levels and O_2_^•−^ production and reduced cAMP levels mediated by AT_1_ receptors [184]. Expression of NOX1, NOX2, and NOX4 (transcript and protein) and NOX activity are increased in aortic VSMC of adult SHR [185]. Basal NOX activity is blocked by the NOX1/NOX4 inhibitor GKT136901 and by NOX1 siRNA in Wistar-Kyoto (WKY) cells, and by siNOX1 and siNOX2 in SHR [179]. Ang II, but not ET-1, upregulates the activity of both NOX1 and NOX2 in the VSMC of SHR, whereas Ang II and ET-1 upregulate only NOX1 in the VSMC of WKY [185]. Transactivation of insulin-like growth factor (IGF-1) receptor by Ang II favors vascular oxidative stress [186]. The aortic media of 32-week-old SHR-SP has marked upregulation NOX1 [185]. The antihypertensive NOX inhibitor apocynin reduces the overexpression of the AT_1_ receptor, NOX2, p47phox, and ROS, reversing the actions of Ang II in conduit and resistance arteries of adult SHR [187]. ARB reduces O_2_^•−^ levels and increases NO bioavailability in the carotid artery and aorta of SHR-SP while reducing BP [188].

Renal superoxide levels in SHR increase, while antioxidant defense systems weaken due to reduced expression and activity of antioxidant enzymes such as ECSOD [189]. Renal cortical expressions of Rac-1, NOX2/O_2_^•−^ and NOX4/H_2_O_2_, p47phox, eNOS, and iNOS are elevated, and NO bioavailability is reduced [190]. The levels of H_2_O_2_ are increased in the renal cortex but not the renal medulla. Additionally, there is no coordinated upregulation or reductions in the activities of CuZn-SOD, EC-SOD, and glutathione peroxidase in the renal cortex and medulla of 12-week-old SHR under basal conditions [178]. These changes indicate increased urinary excretion of 8-isoprostane, thiobarbituric acid reactive substances (TBARS), primarily due to Ang II stimulation of AT_1_ receptors [191,192]. SHR kidneys show excessive O_2_^•−^ production, which reduces NO bioavailability. This effect is normalized by apocynin, an inhibitor of NOX complex assembly and O_2_^•−^ production, the O_2_^•−^ scavenger tempol, and AT_1_ receptor blockade [193]. Endogenous and exogenous H_2_O_2_ upregulates eNOS and nNOS expression in the renal cortex and medulla, although NO activity is reduced due to the quenching effect of ROS [194]. Apocynin, tempol, and ARB normalize NO bioavailability [193]. Increasing NO production by dietary l-citrulline or nitrate supplementation prevents the development of HTN in young SHR between the ages of 4 and 12 weeks [195]. Chronic ARB reduces oxidative stress in the kidneys of 12-week-old SHR and increases glutathione and the activity of SOD, catalase, and glutathione peroxidase [191,196]. More significant increases in oxidative stress are observed in 48-week-old SHR [178].

NOS expression and NO production are elevated in the vasculature and kidneys of young prehypertensive and adult SHR [197,198]. Basal levels of nitrite/nitrate are elevated in the renal cortex and medulla of SHR, and eNOS is upregulated in SHR kidneys [199,200]. Ca^2+−^dependent NOS activity is considerably higher in the renal medulla than in the cortex [199]. Higher levels of nNOS are expressed in the outer medulla and the cortex, whereas iNOS is elevated in the inner and outer medulla and cortex of SHR vs. WKY [201,202]. iNOS is overexpressed in the proximal tubule and reduced in the glomerulus, macula densa, and collecting duct [201,203].

The ability of tempol or apocynin to reduce BP in hypertensive SHR is attributed to a reduction in oxidative stress and the renal excretion of 8-isoprostane. This effect occurs independently of changes in plasma renin activity (PRA), sympathetic nerve activity, and the renal excretion of NO, catecholamines, and ET-1 [204,205]. Overexpression of human heme oxygenase 1 (HO-1) in the kidney (preglomerular arterioles, proximal tubule, and TAL) reduces BP in 4- and 8-week-old SHR [206]. Chronic tempol treatment prevents the BP increase between 6 and 11 weeks of age in SHR with reduced aortic production of O_2_^•−^ in adult SHR [205]. Chronic tempol produces renal vasodilation and reduces renal vascular reactivity to Ang II in 10–12-week-old SHR by reducing NO [207]. A selective increase in renal medullary perfusion is noted with reduced local sensitivity to Ang II [208]. Conversely, tempol increases endogenous H_2_O_2_ production, upregulates renal eNOS and nNOS expression, and reduces NOX activity [209]. Lifelong antioxidant dietary supplementation containing ascorbic acid beginning at a prenatal period delays the onset of HTN. It reduces the severity of HTN in 24-week-old SHR in association with reduced renal cortical NOX2 and p22phox, renal oxidative stress (malondialdehyde and nitrotyrosine abundance), and immunoregulatory calcineurin abundance [193]. The antihypertensive NOX inhibitor apocynin reduces the overexpression of AT_1_ receptor, NOX2, p47phox, and ROS, reversing the actions of Ang II in conduit and resistance arteries of adult SHR [188].

Activation of the RAAS contributes to HTN, inflammation, and nephrosclerosis, which are exacerbated by chronic NO inhibition in adult SHR [210]. Ang II/AT_1_ receptor stimulation of oxidative stress and excess renal NOX-dependent O_2_^•−^ production involves NOX2 and eNOS overexpression and loss of EC-SOD. This loss is responsible for impaired O_2_^•−^ consumption by NO [189,211]. Transient ARB treatment in 4–8-week-old SHR results in a prolonged decrease in BP that lasts up to 48 weeks. This treatment also protects against cardiac and kidney damage, as evidenced by histomorphological changes and reduced albuminuria observed at 72 weeks of age [212]. MR blockade is less effective, whereas transient spironolactone treatment leads to prolonged BP reductions and reduced collagen deposition. In contrast, long-term organ protection is less complete, with no attenuation of glomerulosclerosis and albuminuria at 72 weeks of age [213]. An ACE inhibitor or ARB, administered at non-hypotensive and hypotensive doses, reduces glomerular perivascular collagen deposition without altering the kidney/body weight ratio in young SHR during the development of HTN between 4–12 weeks of age [214]. Implicated as an intrarenal action of RAS independent of a BP change, inhibition of a single RAS component reduces systolic BP in 9-week-old SHR, whereas albuminuria persists [215]. However, combined treatment more effectively reduces albuminuria, glomerular collagen deposition, and tubulointerstitial injury, providing renoprotection to high BP-induced development of CKD [40,215].

Chronic ACE inhibition in weanling SHR prevents the development of HTN [171]. A critical factor is early prevention of the exaggerated vasoconstrictor action of Ang II with significant restoration of normal renal hemodynamics [173]. Consistent with this view, reduced RBF and GFR co-segregate with BP in F2 rats derived from crossbreeding SHR and normotensive WKY [216]. In adult SHR, chronic ACE inhibition alleviates oxidative stress, nephrosclerosis, proteinuria, and HTN, in part by inhibiting the renal cortical advanced end-product (AGE) mediated pathway, including reductions in AGE, RAGE, Ang II, H_2_O_2_, p47phox, NF-kB p65, phosphorylated NF-kB p65, vascular cell adhesion molecule (VCAM)-1 and TGF-β1 [217]. Inhibition of AGE formation also reduces BP and oxidative renal injury in SHR-SP [218]. Intrarenal Ang II activates the AT_2_ receptor to induce natriuresis in normal rats but not in 4- and 12-week-old SHR [219]. In normotensive rats, Ang III increases cGMP that internalizes proximal tubular AT_1_ receptor, NHE3, and Na/K-ATPase, with impaired responses evident before the development of HTN in young SHR [219].

PPARγ agonists are antihypertensive in patients with diabetes or obesity [220]. They prevent the development of HTN and early signs of renal damage in young SHR [221]. Expression of the nuclear transcription factors PPARα and PPARγ is increased in arterial VSMC of 6- and 16-week-old SHR, providing cardiorenal protection with antioxidative and inflammatory actions beyond glycemic and lipidemic control [221]. PPARγ activation inhibits Ang II synthesis and downregulates Ang II-mediated proliferation and apoptosis of mesangial cells of salt-loaded SHR [222]. A BP reduction, renal vasodilation, improved endothelial-mediated vasodilation, and reduced renal vascular reactivity to phenylephrine are produced by PPARγ through decreased expression and activity of GRK-2 (G-protein-coupled receptor kinase-2) in 8-week-old SHR [223]. A PPARγ agonist increases NO availability and RAS, enhances SOD and catalase activity, and elevates Nrf2 gene expression, while also upregulating the density of vasodilator AT_2_ and MAS receptors in the renal cortex of 5-week-old pre-hypertensive SHR [222,224]. Ang (1–7) signaling activates PPARγ and catalase activity while blunting NOX activity in diabetic SHR [225]. Renoprotective PPARγ also increases circulating adiponectin and mitigates renal vasoconstriction induced by Ang II, norepinephrine, and phenylephrine in diabetic SHR. Chronic PPARγ stimulation increases NO bioavailability and COX-2 production of PGI_2_, reducing oxidative stress in adult SHR [226]. In SHR-SP, PPARγ activation exerts antioxidative effects through CuZn-SOD, which occurs independently of reducing BP [226].

ROS stimulates renal sympathetic nerve activity in SHR by activating voltage-gated K^+^ channels [227]. Neonatal sympathectomy reduces renal cortical NOX activity, renal vascular resistance, BP, and salt sensitivity [228,229]. Renal denervation delays the development of HTN and increases urinary Na^+^ excretion in young SHR, suggesting prohypertensive actions of renal efferent nerves on renal hemodynamics and/or tubular reabsorption [230,231]. Selective renal afferent denervation reduces CNS oxidative stress, sympathetic nerve activity, and arterial remodeling in 9-week-old SHR [228]. Tempol causes a more significant reduction in renal sympathetic nerve activity and BP in hypertensive SHR than in normotensive WKY [227,232]. Renal application of tempol reduces O_2_^•−^ activity, inflammation, integrated nerve activity, and renal damage while increasing NO bioavailability [232]. In 10-week-old SHR, renal denervation decreases NOX2 and p47phox expression in the renal medulla, leading to decreased NOX activity in both the renal cortex and medulla [233].

The activation of NF-kB by ROS is crucial in hypertensive-induced renal dysfunction and injury [117]. Activation of pro-inflammatory NF-kB induces cytosolic and mitochondrial oxidative stress and tissue injury, contributing to reduced RBF and GFR in 8-week-old SHR [234]. Chronic NF-kB blockade in SHR between 8 and 15 weeks of age reduces cytosolic and mitochondrial oxidative genes and ROS generation. It also decreases renal interstitial inflammation, attenuates HTN, and normalizes the reduced RBF, GFR, and Na^+^ excretion [235]. Chronic treatment with NAC partially attenuates the rise in BP in young SHR (180 vs. 210 mmHg), decreases ROS and NF-kB expression, and increases NOS activity [236]. Inhibition of RAGE reduces renal injury associated with HTN in adult SHR by reducing RAS activity, NOX-mediated oxidative stress, and NF-kB-mediated inflammation [237]. Renal mRNA levels of *Nox1*, *Nox2*, *p22phox*, and *Ncf1* are also reduced, and this treatment improves renal function (RBF, GFR, albuminuria) and reduces interstitial fibrosis [234].

The anti-aging Klotho gene increases renal expression of Mn-SOD and ET_B_ receptors. Expression of the Klotho gene is downregulated in the kidneys of adult SHR, likely due to overactivity of Ang II and oxidative stress [238]. Klotho supplementation reduces BP, renal angiotensinogen, Ang II, and HIF-1, mammalian target of rapamycin (mTOR), Akt, and medullary fibrosis in adult SHR-SP with nephrosclerosis [239]. Functionally, autoregulation of GFR is restored, and the pressure–natriuresis relation is normalized. ARB reduces oxidative stress, increases Klotho expression, improves the pressure–natriuresis relation, and attenuates the progression of HTN, renal damage, and fibrosis in adult SHR [238]. Renal NOX2 and ET-1 expression and overall NOX activity and O_2_^•−^ production are decreased, as are renal tubular atrophy, tubular obstruction with proteinaceous material, and glomerular collapse (Figure 1) [204,240].

The SHR-SP model represents severe and chronic HTN and stroke. It exhibits sensitivity to salt, increased vascular production of O_2_^•−^, and decreased total plasma antioxidant capacity, all contributing to accelerated age-dependent hypertension compared to SHR [241,242]. Like in SHR, vascular NOX2-derived O_2_^•−^ is vasoconstrictive and negatively impacts NO bioactivity and endothelial function [242,243]. Renal vascular reactivity to agonists is more sensitive and exaggerated in SHR-SP by 4 weeks of age [241]. Albuminuria, juxtamedullary nephron injury, and MCP-1 and TGF-β expression are elevated in 12-week-old SHR-SP fed a high-salt diet for 3 weeks [244]. Tempol, RAAS inhibition, CCB, and vitamins C and E are antihypertensive and renoprotective. ARB has been found to blunt the increase in BP for the first 8 weeks when SHR-SP are fed a high-salt diet [245].

### 4.3. Ang II-Induced Hypertension

#### 4.3.1. The Model

The RAAS is abnormal in normotensive individuals with a positive family history of essential HTN [246]. Ang II does not reduce plasma renin in this population. Subpressor doses produce more significant increases in renal vascular resistance and filtration fraction proportionate to basal BP in untreated adult men with essential HTN, compared to no response in normotensive men. This suggests enhanced renal vascular reactivity, particularly involving the efferent arterioles [247].

In a standard model of experimental HTN, chronic systemic infusion of Ang II at initial suppressor doses leads to a gradual, dose-dependent increase in BP over a week or longer. This increase is accompanied by reduced GFR and Na^+^ excretion and increased tubular reabsorption [248,249]. After that, the Na^+^ balance is attained after increased BP. The primary pathogenic causes involve the prohypertensive intrarenal effects of Ang II and AT1 receptor signaling, which affects the reabsorption and excretion of Na^+^ [250].

Ang II activates AT_1_ receptors to stimulate renal angiotensinogen synthesis, collecting duct (pro) renin receptors, and local renin and Ang II synthesis to increase the distal tubular Na^+^ reabsorption [251]. Genetic deletion of renal-specific AT_1_ receptors markedly reduces the severity of Ang II-induced HTN, as does genetic deletion of renal-specific ACE [74]. In contrast, global deletion of the ACE2 gene and a reduction in vasodilator Ang (1–7) potentiate Ang II-induced HTN in association with increased renal Ang II content [252]. Ang II stimulates the production of NOX-derived ROS that magnify the direct actions of AT_1_ receptor stimulation. NOX1 and NOX2 produce O_2_^•−^ which mediates Ang II-induced oxidative stress, endothelial dysfunction, and exaggerated renal tubular salt transport [78].

#### 4.3.2. Oxidative Stress in the Kidney

Ang II primarily acts through AT_1_ receptors to exert renal anti-natriuretic effects, producing oxidative stress and inflammation, cell growth/proliferation and hypertrophy, cell death, and fibrosis [253]. Downstream signaling targets of Ang II include NOXs, eNOS, iNOS, MAP kinases, RhoA/Rho kinase, transcription factors, protein tyrosine phosphatases, tyrosine kinases, and cytosolic Ca^2+^ [254]. Chronic Ang II infusion increases microvascular ROS and asymmetric dimethylarginine (ADMA). This uncouples eNOS production of NO to that of O_2_^•−^, reduces endothelium-dependent dilation, and switches endothelial function from vasodilation to predominant vasoconstriction associated with HTN [255]. There is a modest level of renal injury in Ang II-induced HTN compared to more severe glomerular and tubulointerstitial injury and proteinuria in L-NAME-induced HTN in rats with similar increases in BP. This difference is mainly because the glomeruli are better protected from fluctuating BP in the Ang II model, which results from a more significant increase in resistance in the kidney’s blood vessels [256]. It is essential to understand that chronic Ang II-induced O_2_^•−^ causes an increase in the RAS in the kidney. This upregulation is characterized by magnified AT_1_ receptor expression in the proximal tubule and (pro)-renin receptor in both the proximal and distal nephron [75]. This represents a positive feedback loop of the intrarenal RAS. Ang II stimulates additional Ang II and ROS formation, magnifying Ang II-induced HTN.

NOX1, NOX2, and NOX4 are upregulated in experimental models [257]. Mice lacking a specific NOX isoform or subunit, either NOX1, NOX2, NOX4, 22phox, p47phox, or NOXA1, exhibit less endothelial dysfunction and less severe HTN induced by acute Ang II infusion [111,258]. An increase in p22phox is required for increased renal NOX activity, NOX1, NOX2, and NOX4 protein expression, oxidative stress, and urinary excretion of 8-isoprostane [259]. Small-interfering RNA targeting p22phox in vivo reveals the role of NOX-derived ROS in the development of HTN produced by Ang II infusion and single nephron GFR by macula densa TGF [259,260]. The p47phox contributes to O_2_^•−^ production by endothelial cells and VSMC and complete expression of Ang II-induced HTN [261]. The magnitude of HTN produced by Ang II infusion is markedly reduced in association with reduced O_2_^•−^ production by endothelium and VSMC in mice lacking p47phox. Superoxide increases renal renin activity, AT_1_ receptor density, and BP [262]. In contrast to the relative short-term Ang II infusion model of HTN, where NOX isoforms are pro-hypertensive, chronic life-long overexpression of renin produces HTN when excess Ang II is present, regardless of whether NOX1 and NOX2 are expressed and active [263,264].

Functionally, acute and chronic Ang II administration increases salt sensitivity by causing renal vasoconstriction and decreasing Na^+^ excretion. This is mediated by oxidative stress, leading to increased O_2_^•−^ generation and reduced NO bioavailability [265]. Acute (3 h) Ang II infusion upregulates Ca^2+^-dependent NOS activity in the renal cortex and medulla without changing eNOS and nNOS protein expression [266]. Ang II/AT_1_ receptor activation stimulates renal cortical mRNA expression of NOX1, p22phox, and p67phox while suppressing that of NOX4 and EC-SOD, responses opposed to AT_2_ receptor activation [267]. Conversely, chronic (3 days) Ang II upregulates Ca^2+^-dependent NOS activity in the renal cortex only, with augmented eNOS and nNOS protein expression in both the renal cortex and medulla [266]. Endothelial dysfunction is linked to decreased NO/cGMP signaling. This is achieved by stimulating PKC-mediated upregulation of NOX1 and NOX2 and uncoupled eNOS production of O_2_^•−^, which quenches NO. Additionally, it results from inhibited eNOS activity, which reduces NO bioavailability [265]. Chronic upregulation of NOX1 by Ang II leads to aortic dissection and aneurysm formation due to increased metalloproteinase 1 protein levels [268]. NOX1 promotes angiogenesis and tumor growth through PPARγ and NF-kB signaling [269]. Aldosterone stimulates the expression of NOX1 in VSMC by activating PKCδ [270]. These effects can be countered by the O_2_^•−^ scavenger tempol in combination with increased blood flow in the inner cortical and medullary regions. This leads to increased excretion of Na^+^ and water while reducing the excretion of 8-isoprostane in animals with Ang II-induced HTN [271]. Vitamin E provides similar renoprotection [271].

Chronic AT_1_ receptor activation increases mRNA expression of NOX1, p22phox, CuZn-SOD, and Mn-SOD in the renal cortex. This leads to increased O_2_^•−^ generation and renal excretion of 8-isoprostane while decreasing renal expression EC-SOD [272]. Additionally, NOX4 expression may be up- or down-regulated. Elevated renal perfusion pressure also increases NOX4 activity in the renal cortex and outer medulla. As a result, eNOS is uncoupled, and NO bioavailability is reduced. Mice lacking NOX1, NOX2, NOX4, or p47phox display less endothelial dysfunction and severe HTN when exposed to acute Ang II infusion.

Ang II-induced oxidative stress increases resistance and remodeling of the preglomerular vasculature, effects implicated in the pathogenesis of renal and cardiovascular disease [273]. Ang II upregulates NOX-derived O_2_^•−^ production while decreasing quenching by NO and SOD. EC-SOD protects against oxidative stress, attenuating renal p22phox expression, NOX activation, the accompanying renal vasoconstriction, and the development of Ang II-induced HTN [274]. CuZn-SOD attenuates afferent arteriolar sensitivity and responsiveness to Ang II and remodeling by reducing O_2_^•−^ and maintaining NO bioavailability; HTN produced by prolonged Ang II infusion is blunted [273]. In CuZn-SOD knockout mice, Ang II-induced high BP, afferent arteriolar media/lumen ratio, O_2_^•−^ production, and vasoconstriction in response to acute Ang II are enhanced. Treatment with SOD mimetic tempol increases arteriole diameter and normalizes the enhanced sensitivity and responsiveness to Ang II in CuZn-SOD deficient mice. Neither SOD1 deficiency nor overexpression affects nitrate/nitrite excretion changes or renal mRNA expression of NO synthase, SOD2/SOD3 isoforms, or Ang II receptors [273].

NO produced by eNOS plays a protective role during the development of Ang II-induced HTN, attenuating renal vasoconstriction and renal injury by minimizing oxidative stress and the inflammation induced by TNF-α [275]. NO inhibition reduces RBF during Ang II infusion [276]. Increased Ang II/AT_1_ receptor activity produces endothelial dysfunction by reducing eNOS/NO activity and uncoupling eNOS to produce O_2_^•−^ [277]. Mice deficient in eNOS respond to chronic Ang II infusion with more severe HTN and renal damage [275]. A SOD mimetic or a TNF-α receptor blocker attenuates the development of HTN and renal injury in eNOS KO mice [116]. Chronic oral administration of sodium nitrite increases vascular cGMP, decreases NOX4 protein content, and reduces ROS production. This prevents HTN and protects against endothelial dysfunction by lowering oxidative stress in Ang II-infused mice [278]. ADMA, a NOS inhibitor, is elevated in Ang II-induced HTN [279].

The NOX subunits p22phox and p47phox contribute to O_2_^•−^ production by endothelial cells and VSMC, leading to the full expression of Ang II-induced HTN [261,280]. Ang II activates vascular p38 MAPK and increases NOX2 expression and O_2_^•−^ production, contributing to increased BP, endothelial dysfunction, and target organ injury in rats with Ang II-induced HTN [281,282]. Ang II/AT_1_ receptor also stimulates p22phox mRNA expression in VSMC [282]. Overexpression of p22phox in VSMC promotes exaggerated vascular reactivity, hypertrophy, and HTN. This occurs through the upregulation of NOX1 and O_2_^•−^ and H_2_O_2_ production. Additionally, eNOS/NO is enhanced in response to increased H_2_O_2_ levels, increasing EC-SOD expression [283]. An increase in p22phox is required for enhanced renal NOX activity, NOX1, NOX2, and NOX4 protein expression, oxidative stress, and urinary excretion of 8-isoprostane [259]. In vivo mRNA silencing of p22phox prevents the development of HTN, reducing protein expression of NOX1, NOX2, and NOX4, but not p47phox, and reducing 8-isoprostane PGF_2α_ excretion [259]. Mice lacking NOX2 or p47phox have weaker responses to Ang II regarding aortic O_2_^•−^ production and medial hypertrophy but not the severity of Ang II-induced HTN [284,285]. NO bioavailability is preserved in renal afferent arterioles of NOX2 null mice, acting to blunt vasoconstrictor responses to Ang II and adenosine [284]. NOX2 overexpression in the endothelium produces exaggerated BP responses to chronic Ang II, endothelial dysfunction, exaggerated vascular remodeling, and HTN [280]. Gp91-tat inhibition of NOX2 and p47phox assembly and activity blocks aortic O_2_^•−^ production and attenuates systolic BP elevation during Ang II-induced HTN [286].

Chronic Ang II elevates renal NOX1-dependent ROS formation and causes proteinuria, DNA damage, and HTN [287]. Genetic deletion of NOX1 abolishes the HTN, aortic O_2_^•−^ production, and vascular proliferation/hypertrophy induced by chronic Ang II infusion [258]. Furthermore, aortic endothelium-dependent cGMP signaling and vasorelaxation in WT mice are impaired following Ang II infusion. In contrast, it is preserved in mice lacking NOX1 [264,288]. Conversely, transgenic overexpression of NOX1 in VSMCs potentiates Ang II-induced HTN [78]. The aortic production of O_2_^•−^ is enhanced, as is medial hypertrophy, and the effects are prevented by tempol. Renal function in wild-type mice is characterized by a transient period of Na^+^ retention during the initial days of Ang II infusion when HTN is initiated [61]. A significant contributor to the development of Ang II-induced HTN is NOXA1/NOX1/O_2_^•−^ stimulation of epithelial sodium channel (ENaC) activity and Na^+^ reabsorption in the distal nephron [61,289].

NOX2 increases mitochondrial O_2_^•−^ and endothelial dysfunction in Ang II-induced HTN [290]. Ang II activates vascular p38 MAPK and increases NOX2 expression and O_2_^•−^ production, contributing to increased BP, endothelial dysfunction, and target organ injury [281]. Overexpression of endothelial NOX2 and ROS production in transgenic mice contribute to endothelial dysfunction, vascular remodeling, and elevated BP in Ang II-infusion HTN [291]. Transfection of the anti-aging gene Klotho attenuates VSMC NOX2 protein expression, O_2_^•−^ production, oxidative stress, and apoptosis in Ang II-induced HTN [292]. Genetic NOX2 deletion reduces O_2_^•−^ generation, renal vasoconstriction, anti-natriuresis, and Ang II-induced HTN while increasing renal nitrite/nitrate and Na^+^ excretion [284]. Mice lacking NOX2 have weaker responses to Ang II in terms of aortic O_2_^•−^ production and medial hypertrophy [284,293], but Gp91ds-tat inhibition of NOX2 also reduces the development of HTN [286].

Contrary to the majority of evidence supporting the importance of NOX1 and NOX2 in short-term Ang II-induced hypertension, a different model of life-long renin overexpression produces HTN regardless of NOX1 or NOX2, possibly because chronically elevated Ang II causes developmental changes [263,264].

Ang II enhances the physical association of the AT_1_ receptor with NOX4, whereas Ang II/AT_1_ receptor activation stimulates NOX4 and p47phox expression, O_2_^•−^ generation, and Gα protein expression and diminishes adenylate cyclase signaling in VSMC [280,294]. Atorvastatin suppresses the magnitude of HTN and reduces vascular membrane translocation of Rac1, NOX4 mRNA, and ROS during chronic Ang II infusion. This effect is even greater when statin treatment is combined with the deletion of NOX1 [295]. Ang II activation of NOX4 and peroxynitrite formation in glomerular mesangial cells causes uncoupling of eNOS and ROS generation, leading to fibronectin synthesis and accumulation of ECM [296]. NOX4 contributes to the ability of Ang II to upregulate AT_1_ receptors in proximal tubular cells. Renal medullary H_2_O_2_ is increased in Ang II-induced HTN in association with Ang II upregulation of AT_1_ receptors and NOX4 [297]. PEG-catalase transiently reduces renal medullary expression of AT_1_ receptors and NOX4 and the magnitude of HTN. Global genetic deletion of NOX4 and the dual pharmacological inhibitor of NOX1/NOX4 GKT137831 attenuate H_2_O_2_ generation, Ang II-induced myofibroblast proliferation and migration, and the severity of Ang II-induced HTN [294,298]. Chronic Ang (1–7) administration reduces NOX4 expression and oxidative stress. It improves eNOS/NO production and kidney function (RBF, GFR, proteinuria) in db/db type 2 diabetic mice [299].

There is a reciprocal relation between ROS and COX2, which increases arterial resistance during Ang II-induced HTN. Inhibition of NOX1, NOX2, or COX2 prevents HTN, abolishes exaggerated vasoconstriction produced by phenylephrine, and restores acetylcholine-induced vasodilation [295]. Antioxidant treatment prevents COX2 stimulation, TxA_2_ synthesis, and vasoconstriction mediated by TP receptor activation [300]. Moreover, COX2 inhibition normalizes O_2_^•−^ and H_2_O_2_ production by preventing increased NOX1 and NOX4 activity [295]. Ang II/AT_1_ receptor stimulation of NOX-derived ROS upregulates glomerular COX-2 expression and activity, as well as PGE_2_ and PGI_2_ production [300]. PGI_2_ signaling activates endothelial NOX4 to promote cytoprotection and angiogenesis [301]. COX2 inhibition and EP_1_ receptor blockade prevent Ang II-induced mesangial cell hypertrophy without affecting PGE_2_-mediated vasodilation [300].

HTN-induced renal injury is associated with stimulation of oxidative stress, inflammation with macrophage infiltration, fibronectin, and TGF-β. Upregulation of renal cytochrome P450 1B1 (CYP1B1) contributes to increased ROS, inflammation, reduced GFR, and renal injury during Ang II-induced HTN [302]. Other changes include renal hypertrophy, proteinuria, heightened reactivity of interlobar arteries to vasoconstrictor agents, increased ERK1/2, p38 MAPK, c-Src signaling, and interstitial fibrosis [303]. An elevated renal perfusion pressure causes renal leucocyte infiltration, capillary rarefaction, and albuminuria through rapamycin/mTOR signaling associated with renal oxidative stress and outer medullary O_2_^•−^ production during Ang II-Induced HTN [304,305]. In contrast, Ang II is responsible for increased renal cortical and outer medullary NOX activity [259].

Upregulation of SODs protects against the effects of elevated Ang II and HTN. Additionally, AT_2_ receptor stimulation counteracts the pro-hypertensive actions of AT_1_ receptor activation, providing protection. Overexpression of EC-SOD protects against oxidative stress and the development of Ang II-induced HTN [274]. This is achieved by reducing renal p22phox expression, NOX activation, and the accompanying renal vasoconstriction and reduced Na^+^ excretion, similar to the beneficial effects of tempol [259]. However, during an Ang II-induced slow pressor response, renal EC-SOD expression is reduced, and, in its absence, renal CuZn-SOD is upregulated to limit excessive Ang II-induced renal oxidative stress [274]. Tempol increases total, inner cortical, and medullary blood flow and excretion of Na^+^ and water while reducing 8-isoprostane excretion in rats with Ang II-induced HTN [306]. The tempol effects are attributed to scavenging O_2_^•−^ since catalase has no additional impact [274]. Treatment of hypertensive rodents with various antioxidants, including vitamins C and E, SOD, tempol, and NOX antagonists, reduces vascular and systemic oxidative stress and BP.

The CCB amlodipine has vasodilatory, antihypertensive, and antioxidant activities that reduce vascular oxidative stress and O_2_^•−^ production, endothelial dysfunction, and aortic hypertrophy in Ang II-mediated cardiorenal injury [307]. The antihypertensive action of CCB is associated with increased endothelial NO bioavailability, reduced ROS production, and renal vascular resistance with improved GFR and Na^+^ excretion [308].

A salt diet influences NOX-dependent oxidant stress and BP responses to chronic Ang II administration [110]. Treatment with Ang II increases mean BP in proportion to salt intake and increases NOX protein expression and oxidative stress [272]. Dietary sodium restriction attenuates organ oxidative stress in Ang II-induced HTN [309]. A low-sodium diet is an antioxidant, reducing aortic H_2_O_2_ production and proteinuria induced by chronic Ang II [310]. HO-1 induction lowers BP and NOX-derived ROS production in the renal medulla of Ang II hypertensive mice [311].

Pro-inflammatory gut microbiota contributes to Ang II-induced HTN and cardiorenal dysfunction [312]. Potentiation of Ang II-induced vascular dysfunction and HTN support NOX2-driven ROS production and iNOS-related endothelial dysfunction, MCP-1/IL-17-driven vascular and kidney immune cell infiltration, and inflammation [313]. Trimethylamine N-oxide (TMAO) potentiates Ang II-induced renal arteriolar vasoconstriction and HTN [314]. Berberine treatment alters gut microbiota composition to reduce plasma TMAO, endothelial dysfunction, and BP [315]. The carbohydrate metabolite sodium butyrate suppresses Ang II-induced HTN by inhibiting the renal (pro)renin receptor and intrarenal RAS [316].

### 4.4. Dahl Salt-Sensitive Hypertension

#### 4.4.1. The Model

Salt-sensitive (SS) HTN often occurs in patients with diabetes, obesity, and CKD who consume excess dietary NaCl [317]. The renal mechanisms involved in developing SS HTN center around the kidneys to excrete the high dietary salt load appropriately [318]. Major factors affecting renal dysfunction and a transient period of Na^+^ retention are vascular and renal oxidative stress and inflammation primarily resulting from activated intrarenal RAAS and SNS [319]. The paradoxical inability of high salt intake to suppress the intrarenal RAAS in SS individuals results in reduced salt excretion for a given BP level [320,321].

A popular experimental model is the development of SS HTN in inbred Dahl rats [322]. Dahl SS rats rapidly develop time-dependent severe HTN and renal injury, glomerulosclerosis, and proteinuria when fed a high salt (4 or 8% NaCl) diet for 2 weeks or more in comparison with more normotensive salt-resistant (SR) genetic control rats [323]. Cross-transplantation of kidneys between SS and SR Dahl rats establishes that genetic differences in host kidney function determine the severity of HTN in recipients of high salt intake [324,325]. After 4 weeks on an 8% NaCl diet, rats with SS donor kidneys become hypertensive with lower RBF and GFR than rats with kidneys from SR rats [326].

#### 4.4.2. Oxidative Stress in the Kidney

Chronic consumption of a high-salt diet causes HTN, renal dysfunction, and injury in Dahl SS but not in normotensive Dahl SR rats [327]. The vasculature and kidneys of Dahl SS HTN rats exhibit oxidative stress with elevated O_2_^•−^ production and reduced SOD protein expression and activity [328]. A high salt diet increases ROS production in the renal cortex, medulla, and F2-isoprostane excretion while downregulating Cu/Zn-SOD and Mn-SOD in the Dahl SS rats (Figure 5) [42]. Expression of cortical NOX1, NOX2, NOX4, p22phox, p47phox, and p67phox mRNA is upregulated. Additionally, there is an increase in NOX activity, production of O_2_^•−^ and H_2_O_2_, and levels of proinflammatory/profibrotic proteins such as COX-2 and PAI-1. Furthermore, activation of the intrarenal RAS occurs, along with increased urinary excretion of H_2_O_2_, TxB_2_, isoprostanes, malonaldehyde, and monocyte/macrophage infiltration [327]. A high salt diet induces vascular hypertrophy, glomerular and interstitial injury, and BP while reducing renal cortical perfusion, plasma H_2_O_2,_ and circulating renin levels [42,329]. There are no compensatory increases in the renal antioxidant enzymes mitochondrial SOD and glutathione peroxidase. Hypertensive Dahl SS rats fed a high-salt diet for 3 weeks exhibited mild renal damage, with more severe injury evident after 5 weeks [327].

Salt sensitivity is linked to oxidative stress, HTN, and ESRD due to upregulation of the intrarenal RAS ((pro)-renin, renin, angiotensinogen, ACE, Ang II, and AT_1_ receptor). Concurrently, systemic PRA is suppressed [254,329]. In turn, increased O_2_^•−^ and reduced NO production upregulate local Ang II/AT_1_ receptors and their activity. Such Ang II activation of AT_1_ receptors contributes to renal oxidative stress, inflammation, proteinuria, glomerulosclerosis, and HTN in Dahl SS rats [330]. Under basal conditions, Dahl SS rats have increased renal levels of H_2_O_2_ due to exaggerated AT1 receptor signaling combined with reduced renal eNOS and upregulated iNOS expression [329]. AT_1_ receptor and NOX1/NOX2/O_2_^•−^ and NOX4/H_2_O_2_/mTORC1 signaling are responsible for pro-hypertensive abnormal renal function and progressive renal injury in salt-induced HTN [42,330]. Exaggerated Ang II/AT_1_ receptor/ROS signaling leads to increased renal cortical COX2 mRNA and protein expression, PGE_2_ excretion, and renal O_2_^•−^ production by NOX1 and NOX2 as well as NOX4-dependent H_2_O_2_ production and activation of the mTORC1 pathway [300]. Thus, oxidative stress plays a significant role in the established phase of HTN and the severity of renal injury. Intrarenal antioxidants CuZn-SOD, Mn-SOD, and glutathione peroxidase show relative decreases [331]. Regulation of NOX activity and reactive oxygen species (ROS) impacts the cellular NAD(P)H/NAD(P+) ratio, which in turn activates NAD(P)H:quinone oxidoreductase 1. This activation helps mitigate high salt/HTN-induced glomerular injury and proteinuria [332]. AT_1_ receptor antagonism is renoprotective, abrogating the effects of Ang II on oxidative stress, intrarenal formation of angiotensinogen and renin, inflammation, vascular hypertrophy, renal dysfunction, proteinuria, and HTN [333,334].

MR activation in prepubertal Dahl SS rats between 4 and 10 weeks of age contributes to high-salt-induced HTN, increased ROS production, inflammation (macrophage infiltration, PAI-1, TGF-β), and renal injury (proteinuria) [335]. During high salt intake, aldosterone upregulates renal NOX4 and p22phox and reduces NO bioavailability, which leads to vascular and renal remodeling and kidney damage in Dahl SS rats [336]. MR antagonism increases CuZn-SOD and Mn-SOD, alleviates glomerulosclerosis and proteinuria, and decreases renal (pro)renin receptor protein expression, angiotensinogen levels, AT_1_ receptor mRNA levels, and kidney Ang II content [335]. Clinical studies indicate that MR antagonism is an effective treatment for SS HTN and idiopathic hyperaldosteronism in humans [337,338].

In Dahl SS HTN, the administration of SOD, tempol, or apocynin reduces BP and O_2_^•−^ levels in both the renal cortex and medulla. This leads to decreased renal injury secondary to reduced vascular production of O_2_^•−^ and the normalization of bioactive NO levels [42,331]. Vitamins C and E reduce renal oxidative stress, inflammation, and BP, improving RBF, GFR, and renal injury [339]. Melatonin prevents kidney injury in SS HTN by decreasing oxidative stress [340]. A high-salt diet causes excessive renal oxidative stress, renal injury, reduced hydrogen sulfide levels, and HTN in Dahl SS rats, which can be inhibited by exogenous hydrogen sulfide and enhanced antioxidant activity [341]. Renoprotection is also provided by activation of NAD(P)H:quinone oxidoreductase 1 that prevents high salt/HTN-induced glomerular injury and proteinuria in Dahl SS rats [332].

Under basal conditions, Dahl SS rats have reduced renal eNOS and upregulated iNOS expression [342]. Dysfunctional renal eNOS and nNOS contribute to Dahl SS HTN, leading to characteristic endothelial dysfunction and ET-1/ET_A_ receptor-mediated renal arterial hypertrophy and glomerulosclerosis [343,344]. ARB reduces oxidative stress and BP, increasing NO and endothelium-dependent vasodilation [345,346]. L-arginine supplementation reduces renal vascular resistance and improves proteinuria in Dahl SS rats [347,348]. Increased NO bioavailability by L-arginine supplementation reduces renal cortical NOX2 and p47phox expression and renal vascular resistance and improves proteinuria in Dahl SS rats [349]. The vasodilator nicorandil increases renal eNOS expression and reduces the uncoupling of eNOS in glomeruli [350]. This vasodilator reduces BP and prevents the development of renal dysfunction in Dahl SS HTN rats. Without blood pressure reduction in SHR, an increase in renal nitric oxide NO provides similar protection against renal injury [351]. Mutated EC-SOD increases O_2_^•−^ and exacerbates endothelial dysfunction in Dahl SS rats consuming a regular salt diet. Genetic deletion of EC-SOD causes CKD characterized by ROS-mediated glomerulosclerosis, proteinuria, focal fibrosis, and necrosis in Dahl SS rats fed a regular salt diet from 8 and 20 weeks of age.

Salt loading downregulates eNOS, but not nNOS, in the aorta and kidney, whereas overall renal cortical NOX activity is upregulated [352]. Dahl rats with SS HTN exhibit endothelial dysfunction and reductions in RBF, GFR, Na^+^ excretion, and pronounced renal injury (glomerulosclerosis and proteinuria) associated with increased renal O_2_^•−^ production and reduced antioxidant capacity [330,331]. In contrast, NOX activity in the renal medulla is minimally reduced compared to more marked suppression in normotensive rats [353]. Relative decreases in the antioxidants CuZn SOD, Mn-SOD, and glutathione peroxidase are observed in both the renal cortex and medulla of Dahl SS rats fed a high-salt diet [331]. Abnormal mitochondrial energy metabolism plays a role in the development of SS HTN; increased oxidative stress and reduced ATP production and SOD are observed in the renal cortex and medulla when fed a high-salt diet [354].

Kidneys of human G protein-coupled receptor kinase 4γ (hGRK4γ) 486V transgenic mice fed a high-salt diet have elevated O_2_^•−^ production in the renal cortex, decreased antioxidant activity, and reduced Na^+^ excretion that contribute to the development of SS HTN [355]. Renal NOX2 is maintained while EC-SOD, CuZn-SOD, Mn-SOD, and eNOS are reduced, and HO-2 is increased.

NOX4 plays a crucial role in the development of salt-sensitive hypertension and renal injury in Dahl rats [356]. Upregulation of NOX4 and H_2_O_2_ production by a high-salt diet activates the mTORC1 pathway that contributes to the established phase of HTN and the severity of renal injury (glomerular damage, albuminuria, and tubular casts) [357]. NOX4/ROS generation enhances salt reabsorption by the medullary thick ascending limb and distal nephron and also reduces medullary blood flow in Dahl SS HTN [358]. Global deletion of NOX4 or the NOX inhibitor apocynin reduces SS HTN and renal injury by decreasing renal H_2_O_2_ generation [332,356].

Activation of NAD(P)H:quinone oxidoreductase 1 is renoprotective, reducing renal NOX activity, ROS production, inflammation injury, proteinuria, and fibrosis in Dahl SS HTN rats [332]. Antioxidant treatment and immune suppression reduce BP and prevent renal dysfunction (reduced RBF and GFR) and damage in Dahl SS HTN [328,359]. A high-salt diet induces several changes that can be reversed through oral L-arginine supplementation. This supplementation increases the endothelial nitric oxide synthase (eNOS) expression and enhances nitric oxide (NO) production while preventing the uncoupling of eNOS from O_2_^•−^ generation. As a result, it restores endothelial function related to vasodilation, providing protective effects distinct from lowering BP [360].

Long-term administration of the SOD mimetic tempol or ARB reduces BP, intrarenal RAS activity (angiotensinogen, Ang II), MAP kinase (ERK1/ERK2, JNK, BMK1) signaling and progressive glomerular proliferative and sclerotic changes, and proteinuria [42]. Tempol causes more significant reductions in renal cortical and medullary O_2_^•−^ production in Dahl SS rats fed high vs. low salt diet. Renal oxidative stress, inflammation, renal injury, and BP are reduced in the Dahl, DOCA-salt, and SHR-SP models of SS HTN by antioxidant vitamins C and E [359].

Renal vasoconstriction and injury, such as glomerular damage, proteinuria, and tubular casts, are improved with L-arginine supplementation. It restores reduced NO synthesis and reduces NOX2 and p47phox expression in the renal cortex of Dahl SS HTN rats, actions that are independent of a BP reduction [347,349]. Chronic inhibition of the serine protease DPP4 activity provides renoprotection, reducing NOX4 expression and oxidation of nucleic acids, lipids, and proteins, albuminuria, attenuates inflammatory agents (NF-*κ*B, TNF*α*, IL-1*β*, IL-6, and MCP-1) and macrophage infiltration [361]. High KCl (2.6%) dietary supplementation decreases BP and protects against renal vasoconstriction and kidney damage in Dahl SS rats despite increasing aldosterone and Na^+^ retention [362]. Atorvastatin is also renoprotective against glomerulosclerosis and proteinuria in SS HTN by reducing NOX/ROS, plasma F2-isoprostane, and TGF-β1 activity and restoring eNOS activity and NO levels [363]. Almandine alleviates high-salt diet-induced HTN and renal dysfunction in Dahl rats by suppressing apoptosis of proximal tubular cells and inhibiting PKC/ROS signaling [334].

High-salt and protein diets modulate the composition of gut microbiota and short-chain fatty acids, directly contributing to the development of SS HTN and renal injury [364,365]. The ketone body beta-hydroxybutyrate attenuates SS HTN and renal damage [366]. Broad-spectrum antibiotics alter gut microbiota and increase SS HTN but decrease BP in SHR, highlighting interactions between individual genetic and microbiota communities [93].

### 4.5. Summary

Animal models, including SHR, Ang II-induced HTN in mice and rats, and Dahl SS rats, demonstrate the connection between the overproduction of ROS, impaired antioxidant defenses, and the onset and progression of HTN. NOXs are primary producers of excess ROS and play a central role in the development and maintenance stages of the HTN. Ang II and high salt intake are major stimuli for exaggerated NOX-derived ROS production and oxidative stress central to the advancement of HTN. High ROS levels directly impact renal function by affecting hemodynamics, vascular reactivity and remodeling, endothelial dysfunction, and tubular transport functions. These processes ultimately influence urinary excretion and BP. Evidence indicates that genetic deletion or targeting of specific NOX isoforms to inhibit ROS production, along with enhancing antioxidant mechanisms such as SOD and catalase to promote ROS scavenging, may improve renal structure and function, lower BP, and ultimately prevent or alleviate the development of HTN and its complications.

## 5. Role of SGLT2 and MR in Chronic Kidney Disease and Hypertension

Experimental evidence shows that ROS can damage cellular and molecular signaling pathways, leading to hypoxia, autophagy, apoptosis, and necrosis, which impact cardiovascular and renal function [367]. Animal and cell culture studies demonstrate that oxidative stress is associated with inflammation, atherosclerosis, HTN, and CKD [368]. Endothelial dysfunction in the early stages of CKD, triggered by increased ROS and inflammation, is associated with atherosclerosis and cardiovascular mortality [369].

### 5.1. Clinical Evidence from Human Studies

In numerous clinical trials, the overall effectiveness of antioxidant strategies has been inconsistent and not very convincing. This includes efforts to enhance the activity of antioxidant enzymes such as SOD or glutathione reductase, the antioxidant vitamins C and E, the polyphenol resveratrol, and the xanthine oxidase inhibitor allopurinol [370]. Human studies provide varying evidence for and against oxidative stress as a causative factor of different forms of HTN [13]. A challenge for future studies is to fully understand the pathophysiologic mechanisms by which oxidative stress affects cells, tissues, and organs at a localized level and how antioxidant approaches reduce cell injury and improve overall health outcomes in HTN and kidney disease.

On the other hand, recent clinical studies provide increasingly consistent and compelling evidence that oxidative stress plays a critical role in the progression of renal dysfunction in diabetic kidney disease (DKD) and CKD [371]. Treatment with pharmacological inhibitors of the sodium-glucose cotransporter 2 (SGLT2) and the MR has improved cardiovascular and renal function in animal and clinical studies [372,373]. Another promising approach is using microRNAs to target genes contributing to oxidative stress [374].

DKD, a rapidly growing cause of CKD, is associated with morbidity, mortality, and cardiovascular disease, including the worsening of HTN [371,372]. Patients with diabetes mellitus are at risk for cardiovascular disease, with about 50% of them developing HTN [375]. HTN, in turn, is a significant risk factor for vascular disease, contributing to accelerated atherogenesis and the progression of diabetic nephropathy [376]. Classical anti-hypertensive therapy is beneficial in slowing the evolution of nephropathy and cardiovascular complications, thereby reducing the risk of cardiorenal disease and mortality [372]. Control of HTN slows the advancement of DKD and its transition to CKD. Preclinical research implicates oxidative stress, inflammation, and increased RAAS activity as central underlying interacting mechanisms in the pathogenesis and progression of kidney disease and HTN. Recent laboratory and clinical evidence highlight a critical pathophysiological role of proximal tubular SGLT2 and tubular MR in both diabetic and non-diabetic forms of kidney disease leading to CKD and HTN [377,378].

Since the 1990s, the progression of DKD has been slowed by specific therapies, such as RAAS blockers (including ACE inhibitors and ARBs), non-steroidal MR antagonists (e.g., finerenone), and SGLT2 inhibitors (e.g., canagliflozin, dapagliflozin, empagliflozin, and tofogliflozin) (Figure 5) [379]. These agents have multiple beneficial effects, including anti-oxidative, anti-inflammatory, anti-fibrotic, and complementary hemodynamic effects, which reduce hyperfiltration and renal stress in patients with DKD. Consequently, their additive benefits slow the progression of renal disease processes [372]. Preclinical findings support the positive effects of this clinical therapy. These observations show that genetic deletion of SGLT2 lowers blood glucose and BP and prevents glomerular hyperfiltration, proteinuria, and macula densa-mediated upregulation of NO formation in type 1 diabetic Akita mice [380].

**Figure 6:**
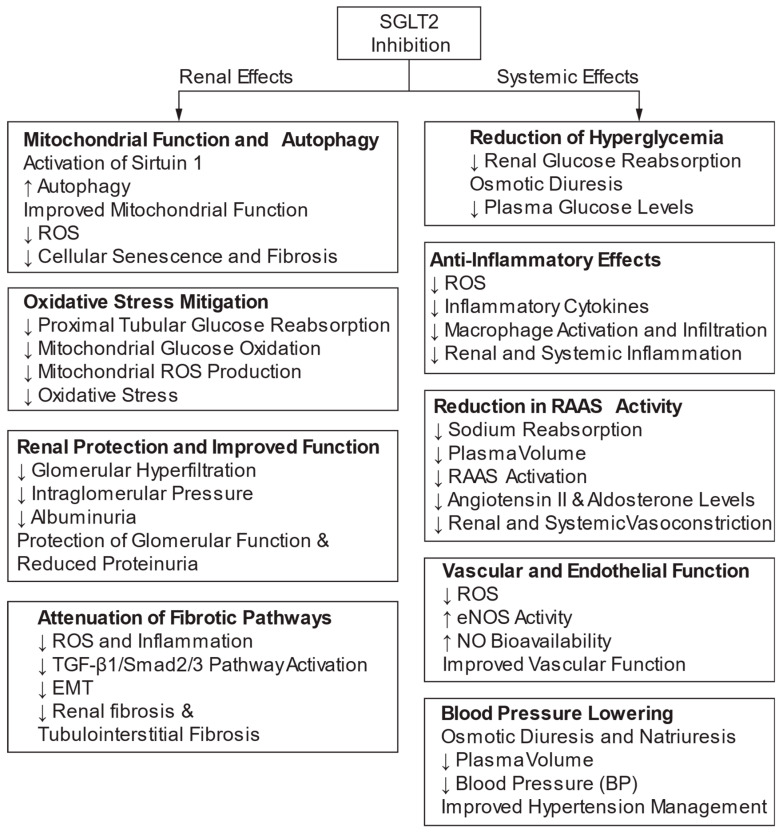
**Renal and systemic effects of proximal tubular SGLT2 (sodium–glucose cotransporter-2) inhibition.** SGLT2 inhibitors lead to glucosuria and promote renal natriuresis by enhancing mitochondrial function and reducing oxidative stress. This improvement helps to enhance glomerular function and decrease tubulointerstitial fibrosis. On a systemic level, SGLT2 inhibition results in lower plasma glucose levels, reduced renal and systemic inflammation, decreased vasoconstriction and vascular injury, Na^+^ retention, and BP.

Proximal tubular SGLT2 inhibitors are anti-hyperglycemic agents that reduce ROS production and help preserve the renal and cardiac function of hypertensive patients, regardless of their type 2 diabetes status [381]. These inhibitors act as osmotic diuretics and possess anti-hypertensive properties by reducing volume retention and BP [378]. The protective effects involve antioxidant, anti-inflammatory, blood glucose-dependent, and -independent mechanisms with little net impact on HbA1C [382]. They may also reduce RAAS activity and sympathetic tone [383]. SGLT2 inhibitors protect the kidney by reducing glomerular capillary HTN and hyperfiltration, limiting physical stress on the filtration barrier and albuminuria (Figure 6) [384,385]. SGLT2 inhibition improves renal oxygenation and function by reducing the oxygen demand for tubular glucose reabsorption [386]. Long-term use of SGLT2 inhibitors improves mitochondrial function and autophagy while attenuating ROS signaling that promotes oxidative stress, inflammation, fibrosis, and senescence (Figure 6) [387]. The preservation of tubular and glomerular function is aided by stimulating erythropoiesis, which enhances organ oxygen delivery [388]. Additionally, the natriuretic effect of SGLT2 inhibition reduces the salt sensitivity of BP [389].

SGLT2 inhibition reduces renal oxidative stress, mitochondrial ROS production, and expression of apoptotic and fibrotic proteins while increasing autophagy during inhibition of the mammalian target of rapamycin in proximal tubular cells of streptozotocin-diabetic mice [390]. Renoprotective SGLT2 inhibition reduces inflammation, angiogenesis, apoptosis, and fibrosis in early-stage diabetic nephropathy in rats with type 2 diabetes [391]. SGLT2 inhibitors attenuate macrophage-mediated renal inflammation and fibrosis by inhibiting monocyte differentiation to macrophages and suppressing activation of inflammasomes and major pro-inflammatory and pro-fibrotic factors [384]. Decreased proximal tubular cell migration and EMT are associated with reduced oxidative stress [392].

SGLT2 inhibition causes vasodilation by preventing oxidative stress-induced inhibition of eNOS, improving NO bioavailability, and reducing endothelial dysfunction through sirtuin 1 activation (Figure 6) [393]. Moreover, these inhibitors help decrease arterial stiffness and levels of pro-fibrotic factors, reducing tubulointerstitial fibrosis [394]. Acute SGLT2 inhibition partially reduces systemic BP by stimulating VSMC voltage-gated K^+^ channels rather than inhibiting Ca^2+^-activated K^+^ channels or ATP-sensitive K^+^ channels [395].

Like SGLT2 inhibitors, glucagon-like peptide-1 (GLP-1) receptor antagonists lower BP and have cardiorenal protective effects in individuals with HTN and diabetes. They reduce oxidative stress and inflammation in the kidneys, other tissues, and organs in in vitro and in vivo models of DKD [396]. Hyperactivation of proximal tubular mTORC1 shifts lipolysis to ketogenesis, preventing reductions in renal ATP levels, tubule-interstitial fibrosis, renal dysfunction, and organ damage [397,398].

Upregulation of the RAAS significantly contributes to the progression of HTN and renal disease [399]. The mineralocorticoid steroid hormone aldosterone, a vital component of the RAAS, is a potential mediator of renal dysfunction and damage in DKD [400,401]. Hyperactivation of the renal MR is associated with increased renal oxidative stress, inflammation, fibrosis, injury, HTN, and progression of DKD/CKD [399].

MR antagonists delay or halt the progression of kidney disease [10]. Blocking MR receptor activation in endothelium, smooth muscle cells, podocytes, tubular cells, inflammatory cells, and fibroblasts can counteract harmful effects on kidney structure and function [402]. MR antagonists can alleviate or even stop the transition from acute kidney injury to diabetic nephropathy and CKD [403]. Besides pharmacological antagonists, micro RNAs and small noncoding RNA molecules are under investigation to suppress MR expression and signaling, attenuating proteinuria and renal injury in DKD [404]. Reducing oxidative stress, inflammation, and fibrosis and improving renal vascular and tubular function are key cellular/molecular mechanisms contributing to cardiovascular and renal health [401].

### 5.2. Animal Experimental Models of Hypertension

Chronic SGLT2 inhibition reduces BP in adult SHR. Transcriptome analysis shows that this inhibition exerts a reno-protective effect in SHR by lowering inflammation and functioning through lysosomal, phagosomal, and autophagic pathways [405,406]. In addition to enhancing immunity and controlling inflammation, SGLT2 treatment also reduces oxidative stress and dysregulation of lipid metabolism.

SGLT2 inhibition lowers BP in Ang II-induced HTN, partly by decreasing intrarenal RAAS activity [407,408]. Reductions in ROS production, inflammation, and sympathetic nerve activity also contribute to cardiorenal protection from injury [409].

Chronic activation of the Ang II/AT_1_ receptor affects the expression and activity of redox-sensitive SGLT2 in proximal tubular cells through increased ROS production and oxidative stress [410]. Upregulated SGLT2 reduces GFR, leading to proteinuria, endothelial dysfunction, and senescence. This is caused by microparticle-induced NOX-driven oxidative stress, which reduces eNOS and NO production [411]. Also reduced are pro-atherosclerotic, pro-fibrotic, and pro-remodeling responses in macro- and micro-vessels, which may be independent of BP changes [409]. Pharmacological SGLT2 inhibition lessens Ang II-induced renal injury, including glomerulosclerosis, albuminuria, and tubulointerstitial fibrosis [412].

Chronic SGLT2 inhibition or MR antagonism provides cardiorenal protection in renin transgenic TGR(mRen2)27 rats during chronic NO inhibition. Systolic BP, renal vasculopathy, proteinuria, and fibrosis are reduced [413].

Polymorphisms in the SGLT2 gene are associated with the sensitivity of BP to salt and the incidence of HTN in Chinese adults [414]. In nondiabetic Dahl SS hypertensive rats, chronic inhibition of the proximal tubular SGLT2 produces glucosuria and natriuresis, leading to reduced BP and protection against kidney dysfunction, regardless of gender [415]. An underlying mechanism involves reducing the pressure–natriuresis relation without altering the RAAS or expression/activity of major Na^+^ transporters along the nephron [377]. The pressure–natriuresis relation is normalized to a lower BP associated with increased renal medullary HIF-1α expression and reduced inflammation [416]. The BP-lowering effect of SGLT2 inhibition also appears to involve attenuating Ca^2+^ uptake in VSMC through TRIPC3 channels [417]. SGLT2 inhibition attenuates salt-sensitivity of BP in association with reduced sympathetic nerve activity driven by excessive brain oxidative stress [418].

The anti-hypertensive and renoprotective effects of SGLT2 inhibition are linked to changes in Na^+^ balance in Dahl SS HTN [377]. In addition, it affects total body Na^+^ levels and decreases glomerulosclerosis, tubulointerstitial fibrosis, and urinary protein excretion. SGLT2 inhibition and ARB result in natriuresis associated with reduced renal expression of the Na^+^ transporters NH3 and NKCC2 [419].

As a renal protector, SGLT2 inhibition ameliorates renal inflammation and pro-fibrotic EMT, reducing HTN-associated renal injury in Dahl SS HTN [420]. This is achieved by reducing oxidative stress and restoring sirtuin 3/FOXO3a/catalase signaling. Additionally, renal fibrosis is blunted by the TGF-β1/Smad2/3 pathway suppression [421]. When SGLT2 inhibitors are combined with ARB or ACE inhibition, the anti-hypertensive and anti-fibrotic effects in Dahl hypertensive rats are more pronounced [422].

MR blockade or SGLT2 inhibition reduces renal oxidative stress, NOX2 and NOX4 mRNA, and mitochondrial dysfunction-associated collagen deposition and fibrosis in Dahl SS rats [423]. MR antagonism exerts cardioprotective actions, reducing oxidative stress, inflammation, BP and cardiac dysfunction, remodeling, and fibrosis [413]. SGLT2 inhibition reduces systolic BP, cardiac hypertrophy, and perivascular fibrosis in Dahl rats fed a high-fat diet [424].

### 5.3. Summary

Experimental evidence shows ROS can damage cellular and molecular signaling pathways, leading to cardiovascular and renal dysfunction. Despite inconsistent results of antioxidant therapy in humans, recent clinical studies support the role of oxidative stress in progressive renal dysfunction in DKD and CKD. SGLT2 inhibitors and MR antagonists have improved cardiovascular and renal function by reducing oxidative stress, inflammation, and RAAS activity. Combined with ARBs or ACE inhibitors, these therapies provide additional renal and antihypertensive protection, effectively reducing proteinuria, fibrosis, and blood pressure (BP). SGLT2 inhibitors also lower BP by balancing Na^+^ and improving renal oxygenation, making them promising therapeutics in managing CKD and HTN.

## 6. Conclusions

Understanding the interactions between genetic and environmental factors is critical for developing targeted interventions to manage renal dysfunction and the development and maintenance of HTN. Personalized treatments targeting specific genetic pathways may benefit hypertensive patients by reducing and reversing kidney damage. Future antioxidative and anti-inflammatory treatments may focus on genetic pathways, the renal RAS, and gut microbiota to manage HTN and its renal complications. Targeting oxidative stress by inhibiting specific NOX isoforms and enhancing antioxidant mechanisms can improve renal function, electrolyte excretion, and lower BP. Animal models remain essential in understanding the pathogenesis of HTN and testing novel therapeutic approaches. There is consistent evidence supporting oxidative stress as an essential pathological mechanism in the initiation and progression of renal dysfunction. SGLT2 and MR pharmacological inhibitors exert multiple actions that can improve cardiovascular and renal function. Combining different pharmacological agents may offer additional benefits in slowing the progression of renal disease and HTN. Future research should focus on individualized treatment approaches based on genetic and environmental factors, particularly those alleviating the harmful effects of oxidative stress.

## Abbreviations

HTN	Hypertension
BP	Blood pressure
ROS	Reactive oxygen species
CKD	Chronic kidney disease
ESRD	End-stage renal disease
RAAS	renin–angiotensin–aldosterone system
SGLT2	Sodium–glucose cotransporter 2
NOX	NADPH oxidase
MR	Mineralocorticoid receptor
SS HTN	Salt-sensitive hypertension
GFR	Glomerular filtration rate
SHR	spontaneously hypertensive rats
Ang II	Angiotensin II
SNS	Sympathetic nervous system
NO	Nitric oxide
ET-1	Endothelin-1
TxA2	Thromboxane A2
PKC	Protein kinase C
MAPK	Mitogen-activated protein kinase
VSMC	Vascular smooth muscle cells
ECM	Extracellular matrix
TGF-β1	Transforming growth factor beta 1
EMT	Epithelial-mesenchymal transition
DKD	Diabetic kidney disease
ARBs	Angiotensin receptor blockers
